# Complete mitochondrial genome of *Syzygium samarangense* reveals genomic recombination, gene transfer, and RNA editing events

**DOI:** 10.3389/fpls.2023.1301164

**Published:** 2024-01-09

**Authors:** Guilong Lu, Qing Li

**Affiliations:** ^1^ Institute of Vegetables, Tibet Academy of Agricultural and Animal Husbandry Sciences, Lhasa, China; ^2^ College of Horticulture and Landscape Architecture, Henan Institute of Science and Technology, Xinxiang, China

**Keywords:** *Syzygium samarangense*, mitogenome, gene loss, evolution analysis, RNA editing

## Abstract

Wax apple (*Syzygium samarangense*) is a commercial fruit that belongs to one of the most species-rich tree genera in the world. We report here the first complete *S. samarangense* mitogenome obtained using a hybrid assembly strategy. The mitogenome was a 530,242 bp circular molecule encoding 61 unique genes accounting for 7.99% of the full-length genome. Additionally, 167 simple sequence repeats, 19 tandem repeats, and 529 pairs of interspersed repeats were identified. Long read mapping and Sanger sequencing revealed the involvement of two forward repeats (35,843 bp and 22,925 bp) in mediating recombination. Thirteen homologous fragments in the chloroplast genome were identified, accounting for 1.53% of the mitogenome, and the longest fragment was 2,432 bp. An evolutionary analysis showed that *S. samarangense* underwent multiple genomic reorganization events and lost at least four protein-coding genes (PCGs) (*rps2*, *rps7*, *rps11*, and *rps19*). A total of 591 RNA editing sites were predicted in 37 PCGs, of which *nad1*-2, *nad4L*-2, and *rps10*-2 led to the gain of new start codons, while *atp6*-1156, *ccmFC*-1315 and *rps10*-331 created new stop codons. This study reveals the genetic features of the *S. samarangense* mitogenome and provides a scientific basis for further studies of traits with an epistatic basis and for germplasm identification.

## Introduction

Wax apple (*Syzygium samarangense*, also known as java apple, rose apple, wax jambu, and bell fruit) is a small evergreen tree in the family Myrtaceae that is native to the Andaman and Nicobar islands and to the Malaysian Archipelago (Khandaker and Boyce, 2016; Banadka et al., 2022). The genus is extremely rich in germplasm resources, with 1,193 recognized species worldwide. It is mainly grown in tropical and subtropical regions and is distributed from Africa to India, across Southeast Asia, and extending to Hawaii in the Pacific (Govaerts et al., 2008). Wax apple fruit is rich in proteins, dietary fiber, sugar, vitamins, flavonoids, phenolic acids, and other nutrients. Phenolic acids antioxidant, anti-cancer, anti-bacterial, anti-diabetic, and other biological activities (Banadka et al., 2022). In addition, the extracts of leaves, flower buds, bark, and roots of wax apple are effective in removing fire and toxins, drying dampness, and relieving itching (Chinese Herbalism Editorial Board, 1999; Gurib-Fakim, 2006). The species also has high ornamental value, mostly owing to its beautiful shape, fragrant and beautiful flowers, bright fruit color, beautiful fruit shape, and long hanging period. In recent years, the rapid development of the wax apple industry has increased the demand for high quality, high yield, and high resistance varieties. Genome and transcriptome research on wax apple has laid a scientific foundation for the utilization of excellent germplasm resources and improvement of varietal traits (Chen et al., 2017; Liu et al., 2018; Low et al., 2022).

Mitochondria, which provide energy for various physiological activities, are important organelles in eukaryotic cells (Møller et al., 2021). Originally as an endosymbiotic form of alphaproteobacteria (Lang et al., 1999), mitochondria are semi-autonomous organelles exhibiting predominantly matrilineal inheritance (Greiner et al., 2015). Previous studies have shown that mitochondria play an important role in plant growth and development and are closely associated with growth vigor, chloroplast function (Chevigny et al., 2020), stress tolerance (Liberatore et al., 2016). Compared to animal mitogenomes, plant mitogenomes exhibit complex and unique genetic features. Their structures are remarkably diverse, with circular, linear, highly branched, sigma-like, and networked types; and the genome sizes vary widely among species, ranging from 66 Kb (*Viscum scurruloideum*) (Skippington et al., 2015) to 11.7 Mb (*Larix sibirica* Ledeb.) (Putintseva et al., 2020). During long-term evolution, the number of mitochondrial genes in extant plants is very low, and most of them have been transferred into the nucleus. There are generally 32-67 genes that are sparsely distributed and have a highly conserved coding sequence, with a large number of repetitive sequences distributed among them (Mower et al., 2012; Richardson et al., 2013). Accumulation of repetitive sequences may lead to frequent recombination events and structural variation in the mitogenome (Sloan, 2013; Wu et al., 2022), whereas active genomic rearrangements can even lead to cytoplasmic male sterility (Kubo et al., 2011; Liao et al., 2020; Wang et al., 2022). The plant mitogenome can integrate plastome and nuclear DNA fragments and even exogenous mitogenomes, which is a major driver of the enlargement and rapid evolution of plant mitogenomes (Rice et al., 2013; Prasad et al., 2022). In addition, RNA editing events are widespread in plant mitochondrial transcripts, where they are critical for the production of functional proteins and adaptive evolution (Gommans et al., 2009; Ichinose and Sugita, 2017) and are closely associated with cytoplasmic male sterility (Gallagher et al., 2002; Wei et al., 2010). In summary, plant mitogenomes have become an important tool for species classification, evolutionary analyses, and parental traceability (Sibbald et al., 2021; Wang et al., 2022; Zhang et al., 2023). However, related research lags far behind studies on chloroplast and plastid genomes owing to the complexity of the mitogenome. The complete mitochondrial genome of *S. samarangense* has not been reported in public databases (https://www.ncbi.nlm.nih.gov/genome/browse/#!/organelles/).

In this study, the main *S. samarangense* cultivar in China, Black Diamond (Zheng, 2011), was selected for the following investigations: 1) the mitogenome was assembled, annotated, and comparatively analyzed; 2) the repetitive sequences present in this genome were identified, and the possible structure of the repeat-mediated genomic recombination was verified using Sanger sequencing and polymerase chain reaction (PCR) validation; 3) the chloroplast genome of the cultivar was assembled, and the transfer of homologous fragments between this genome and the mitogenome was identified; 4) phylogeny and collinearity analyses were conducted to identify the relationships between this species and closely related species; and 5) RNA editing events were predicted to be present in the protein-coding gene (PCG) transcripts, and editing sites that could create new start and stop codons were validated. This study aims to provide a scientific and theoretical basis for the in-depth understanding of the genetic characteristics of *S. samarangense* and further germplasm identification.

## Materials and methods

### Plant material, DNA and RNA preparation, and sequencing

The young leaves of wax apple (Black Diamond) were harvested from the National Agricultural Science and Technology Park, located in Lhasa, Tibet Autonomous Region, China (Coordinates: 91°2’8’’E, 29°38’15’’N; Altitude: 3650 m). The gDNA and RNA were separately extracted using the TianGen Plant Genomic DNA Kit and the RNAprep Pure Plant Kit, respectively (Beijing, China). The NanoDrop One Microvolume UV-Vis Spectrophotometer (Thermo Fisher Scientific, Massachusetts, USA) was used to ensure the integrity of the samples. Samples meeting quality thresholds were then sent to Wuhan Benagen Tech Solutions Company Limited (Wuhan, China) for sequencing, after packing in dry ice. Using DNBSEQ-T7 (developed by Shenzhen Huada Intelligent Technology Co., Ltd., Shenzhen, China), short reads were sequenced, followed by a quality control filtering process on the raw reads using fastp v0.21.0 (Chen, 2023). The Nanopore PromethION sequencer from Oxford Nanopore Technologies (Oxford, UK) was employed to sequence long reads, and NanoFilt v2.8.0 (De Coster et al., 2018) was used for quality control. Long non-coding RNA (lncRNA) sequencing was performed via MGISEQ-2000 (by Shenzhen Huada Intelligent Technology Co., Ltd., Shenzhen, China), and the raw reads were subjected to quality control filtering using SOAPnuke v2.0 (Chen et al., 2018).

### Mitogenome assembly

Initially, Flye v2.9.2 software (University of California, San Diego, USA) (Kolmogorov et al., 2019) was utilized to assemble the long-read sequencing data. Following this, the *Arabidopsis thaliana* mitogenome was used as a reference sequence to identify contig fragments containing mitochondrial genomes via the BLASTn program. Both long-read and short-read data were then aligned to mitogenome contigs using BWA v0.7.17 (Li and Durbin, 2010), after which matched reads were filtered, extracted, and stored separately for subsequent assembly. In the final step, short-read and long-read sequencing data were merged using Unicycler v0.4.7 (The University of Melbourne, Victoria, Australia) (Wick et al., 2017), facilitating hybrid assembly with parameters “- -kmers 57,67,77”, and visualized graphics were obtained using Bandage v0.8.1 (Wick et al., 2015).

### Mitogenome annotation and analysis

GeSeq v2.03 (https://chlorobox.mpimp-golm.mpg.de/geseq.html) (Tillich et al., 2017) was utilized to annotate PCGs of the *S. samarangense* mitogenome, with the mitogenomes of *A. thaliana* (NC_037304), *Liriodendron tulipifera* (NC_021152.1), and *Rhodomyrtus tomentosa* (NC_071968.1) used as reference genomes. tRNA and rRNA genes were annotated using tRNAscan-SE v.2.0.11 (Chan et al., 2021) and BLASTN v2.13.0 (Chen et al., 2015), respectively, and errors in annotation were manually fixed using Apollo v1.11.8 (Dunn et al., 2019). For the extraction of protein-coding gene sequences of the *S. samarangense* mitogenome, PhyloSuite v1.2.2 (Xiang et al., 2023) was employed. Mega software (Tamura et al., 2021) was utilized for the codon preference analysis of PCGs and the calculation of the relative synonymous codon usage values (RSCU). RSCU > 1 signifies that the codon is preferentially used by amino acids, whereas RSCU < 1 denotes the opposite.

### Mitogenome repeat elements

The simple sequence repeats (SSRs) were identified using MISA v2.1 (https://webblast.ipk-gatersleben.de/misa/) with the parameter “1-10 2-5 3-4 4-3 5-3 6-3” (Beier et al., 2017). Tandem repeats were recognized using TRF v4.09 (https://tandem.bu.edu/trf/trf.unix.help.html) with the parameter “2 7 7 80 10 50 500 -f -d -m” (Behboudi et al., 2023). Interspersed repeats were detected using the REPuter web server (https://bibiserv.cebitec.uni-bielefeld.de/reputer/) (Kurtz and Schleiermacher, 1999) with the minimum repeat size set at 30 bp.

### Validation of repeat-mediated recombination

To confirm the proposed genomic structure of the *S. samarangense* mitogenome, each set of repeats and their sequences extending 500 bp upstream and downstream were extracted as reference points. Primer-BLAST (https://www.ncbi.nlm.nih.gov/tools/primer-blast) was used to design primers for the four pathways of the dual bifurcation structure, and PCR amplification along with Sanger sequencing (You et al., 2023) was used to verify the validity of the junction sequences. The PCR amplification procedure was carried out in a 50 μL total volume, containing 2 μL of gDNA template, 2 μL each of upstream and downstream primer (10 μmol/L), 25 μL of 2× Rapid Taq Master Mix (Vazyme Biotech Co., Ltd., Nanjing, China), and 19 μL of ddH_2_O. The PCR steps involved a 95°C pre-denaturation step for 3 min, 35 cycles of 95°C denaturation for 15 s, 55°C annealing for 15 s, and 72°C extension for 30 s, and a final 72°C extension for 15 min.

### Chloroplast genome assembly and gene transfer analysis

GetOrganelle v1.7.7.0 (Freudenthal et al., 2020) was used to assemble the *S. samarangense* chloroplast genome with parameters set to “-R 15 -k 21,45,65,85,105 -F embplant_pt”. The chloroplast genome was then annotated with CPGAVAS2 (Liu et al., 2012) and the annotation results were corrected using CPGView software (Liu et al., 2023). Homologous fragments in the mitochondrial and chloroplast genomes were examined using BLASTN v2.13.0 (Chen et al., 2015) with an e-value threshold of 1*e*-5. The results were visualized via Excel v2021 and Circos v0.69-9 (Zhang et al., 2013).

### Phylogenetic and collinearity analysis

The mitogenomes of 30 species closely related to *S. samarangense* were downloaded from NCBI for a phylogenetic analysis (**Supplementary Table 1**), with *Tribulus terrestris* (MK431825.1) and *Zygophyllum fabago* (MK431827.1) of Zygophyllales assigned as outgroups. PhyloSuite software was used to extract shared genes in the mitogenomes. A multiple sequence alignment was generated using MAFFT v7.505 (Katoh et al., 2019). The “GTR +F+I+I+R2” model was selected for the phylogenetic analysis using IQ-TREE v2.1.3 (Minh et al., 2020), and the maximum likelihood tree was visualized using iTOL v6 (https://itol.embl.de/) (Letunic and Bork, 2007). The mitogenome sequence information for *S. samarangense* and seven closely related species within the same order were analyzed via BLASTn. Homologous sequences exceeding 500 bp were maintained as conserved co-linear blocks, and a multiple synteny plot was drafted using MCscanX (Tang et al., 2008).

### Identification and validation of RNA-editing sites

By using TopHat2 (Mehmood et al., 2020), mitochondrial DNA sequences from the *S. samarangense* mitogenome were mapped to transcriptomic data to obtain transcripts. The potential RNA editing sites were identified by REDItools v2.0 (Flati et al., 2020) by a comparison of DNA and RNA sequences and the selection of positions with a coverage depth ≥ 100× and editing frequency ≥ 0.1. Primer-BLAST was used to design primers for the RNA editing sites that generate start and stop codons (**Supplementary Table 2**). A HiScript III 1st Strand cDNA Synthesis Kit (Vazyme, Nanjing, China) was employed to convert the extracted wax apple RNA into cDNA. Both gDNA and cDNA were used as templates for PCR amplification, employing the same system and procedure described in the validation of repeat-mediated recombination. To confirm the RNA editing sites, the amplified products were sequenced and compared.

## Results

### Mitogenome assembly, gene annotation, and analysis

**Figure 1:**
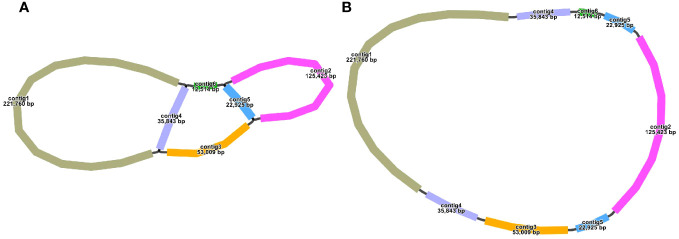
Mitogenome sketch **(A)** and master circular structure **(B)** in *S. samarangense*.

The *S. samarangense* mitogenome was assembled using 10.39 Gb short reads and 11.23 Gb long reads by a hybrid assembly strategy. It contained six contigs, including two double bifurcating structures (**Figure 1A**). The longest contig (221,760 bp) and the shortest contig (12,514 bp) were single-copy regions. The repetitive sequences were determined based on long-read data and one master circular structure was obtained (**Figure 1B**) with a total length of 530,242 bp (27.43% Adenine, A; 27.53% Thymine, T; 22.63% Cytosine, C; and 22.41% Guanine, G). The size of this genome differs slightly from those of other species of the same family: *Rhodomyrtus tomentosa* (400,547 bp, NC_071968.1), *Eucalyptus grandis* (478,813 bp, NC_040010.1), and *E. polybractea* (580,440 bp, CM048836.1).

**Figure 2:**
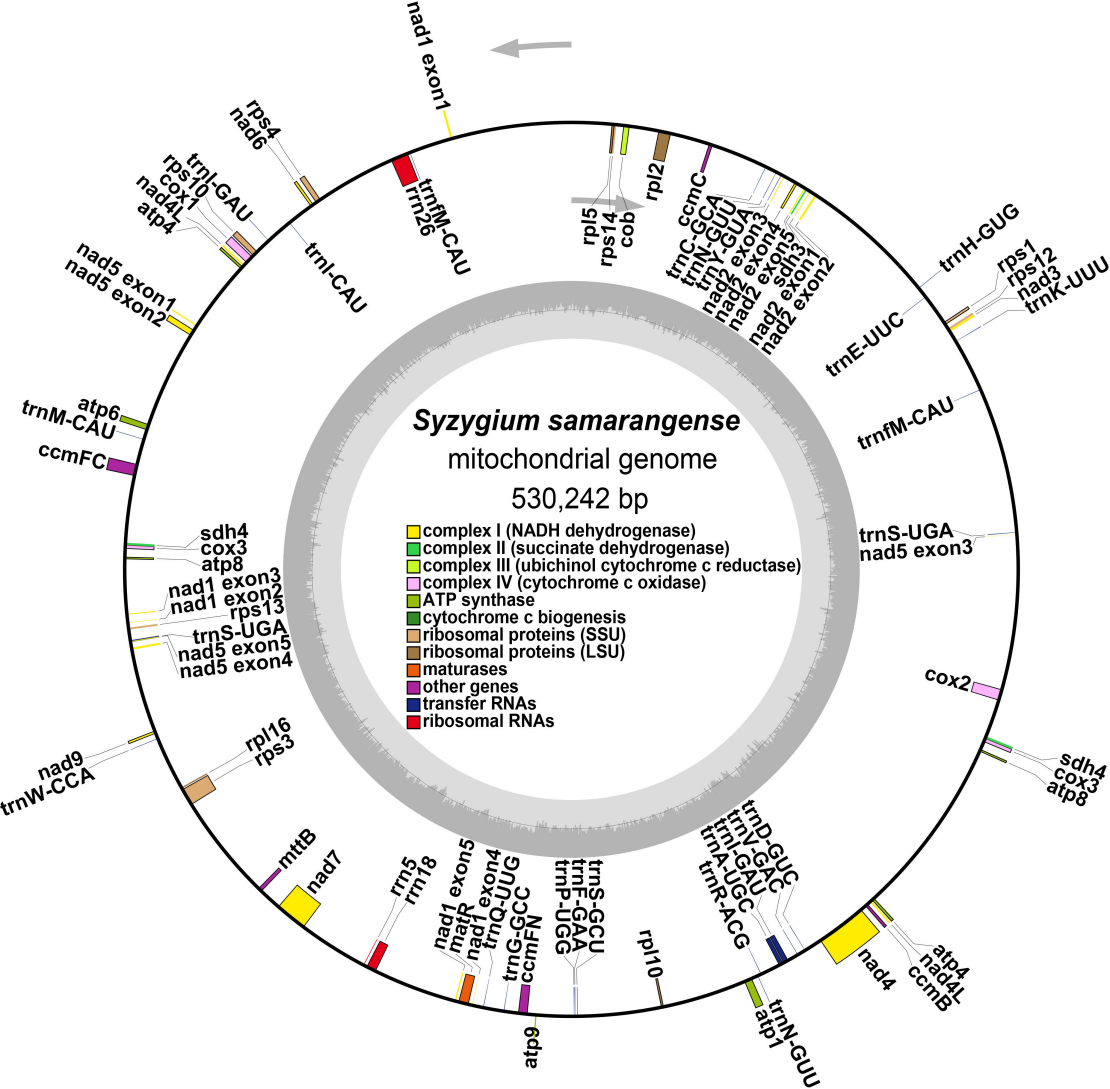
Circular maps of the *S. samarangense* mitogenome.

The *S. samarangense* mitogenome was annotated with 37 unique PCGs, including 24 core genes and 13 non-core genes, 21 tRNA genes (four tRNAs were multiple copies), and three rRNA genes (**Figure 2**, **Table 1**). *S. samarangense* lost four genes (*rps2*, *rps7*, *rps11*, and *rps19*) compared to the PCGs of the “fossilized” *L. tulipifera* mitogenome (Richardson et al., 2013). The total length of coding sequences in the *S. samarangense* mitochondrial genome (PCGs, tRNA, and rRNA genes) was 42,391 bp. This accounted for 7.99% of the entire genome, while over 90% belonged to intergenic regions.

**Table 1:** Genes in the *S. samarangense* mitogenome.

Group of genes		Name of genes
Core genes	ATP synthase	*atp1*, *atp4* (×2), *atp6*, *atp8* (×2), *atp9*
	NADH dehydrogenase	*nad1*, *nad2*, *nad3*, *nad4*, *nad4L* (×2), *nad5*, *nad6*, *nad7*, *nad9*
	Cytochrome b	*cob*
	Cytochrome c biogenesis	*ccmB*, *ccmC*, *ccmFC*, *ccmFN*
	Cytochrome c oxidase	*cox1*, *cox2*, *cox3* (×2)
	Maturases	*matR*
	Protein transport subunit	*mttB*
Variable genes	Ribosomal protein large subunit	*rpl2*, *rpl5*, *rpl10*, *rpl16*
	Ribosomal protein small subunit	*rps1*, *rps3*, *rps4*, *rps10*, *rps12*, *rps13*, *rps14*
	Succinate dehydrogenase	*sdh3*, *sdh4* (×2)
rRNA genes	Ribosome RNA	*rrn5*, *rrn18*, *rrn26*
tRNA genes	Transfer RNA	*trnA-UGC*, *trnC-GCA*, *trnD-GUC*, *trnE-UUC*, *trnF-GAA*, *trnfM-CAU* (×2), *trnG-GCC*, *trnH-GUG*, *trnI-CAU*, *trnI-GAU* (×2), *trnK-UUU*, *trnM-CAU*, *trnN-GUU* (×2), *trnP-UGG*, *trnQ-UUG*, *trnR-ACG*, *trnS-GCU*, *trnS-UGA* (×2), *trnV-GAC*, *trnW-CCA*, *trnY-GUA*

The number in brackets represents the copy number of the gene.

RSCU, which reflects the optimized results of the evolution of plant ecological adaptations, is the “dialect” of a gene or genome (Tyagi et al., 2023). The amino acids encoded by 37 PCGs from the *S. samarangense* mitogenome generally showed codon usage bias, except for the RSCU of 1.00 for the initiation codon (AUG) and tryptophan (UGG) (**Supplementary Figure 1**, **Supplementary Table 3**). For example, alanine (Ala) had a high codon usage preference for GCU with the highest RSCU value of 1.56. Histidine (His) had a usage preference for CAU, while tyrosine (Tyr) had a usage preference for UAU, both of which had an RSCU value of 1.52. In addition, cysteine (Cys), lysine (Lys), phenylalanine (Phe), and valine (Val) had maximum RSCU values below 1.20 and did not have strong codon usage preference. However, the third codon of these amino acids has a greater preference for the use of U or A than other nucleotides.

### Repeat sequence analysis and repeat-mediated recombination

**Figure 3:**
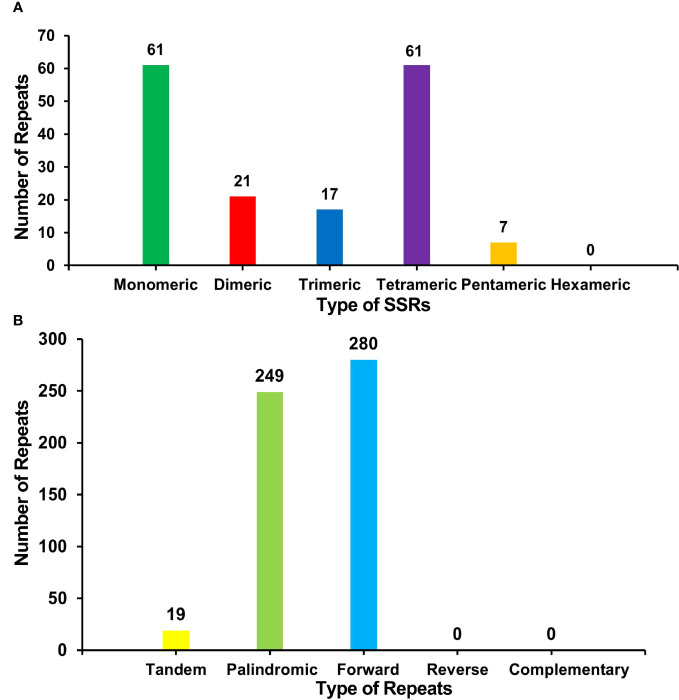
**(A)** Type and number of SSRs in the *S. samarangense* mitogenome. Green, red, blue, purple, and yellow indicate monomeric, dimeric, trimeric, tetrameric, and pentameric, respectively. **(B)** Type and number of repeat sequences in the *S. samarangense* mitogenome. Yellow, light green, and blue indicate tandem repeats, palindromic repeats, and forward repeats, respectively.

There were many repetitive sequences in the *S. samarangense* mitogenome (**Supplementary Figure 2**). A total of 167 simple sequence repeats (SSRs) were identified throughout the genome (**Figure 3A**, **Supplementary Table 4**), and monomeric and dimeric forms of SSRs accounted for 49.10% of the total SSRs. T monomeric repeat sequences (No.35) accounted for 57.38% of the 61 monomeric SSRs, and the TA repeat was the most common type of dimeric SSR, accounting for 38.10% of dimeric SSRs; no hexamer SSRs were detected. Nineteen tandem repeats with lengths between 15 bp and 33 bp and ≥76% match were identified (**Supplementary Table 5**). In addition, 529 pairs of dispersed repeats with ≥30 bp were detected (**Figure 3B**, **Supplementary Table 6**), including 249 pairs of palindromic repeats, 280 pairs of forward repeats, and no reverse and complementary repeats. The total length of the above three types of SSRs, tandem repeats, and interspersed repeats were 1,951 bp, 799 bp and 177,672 bp, accounting for 0.37%, 0.15%, and 33.51% of the mitogenome, respectively.

**Figure 4:**
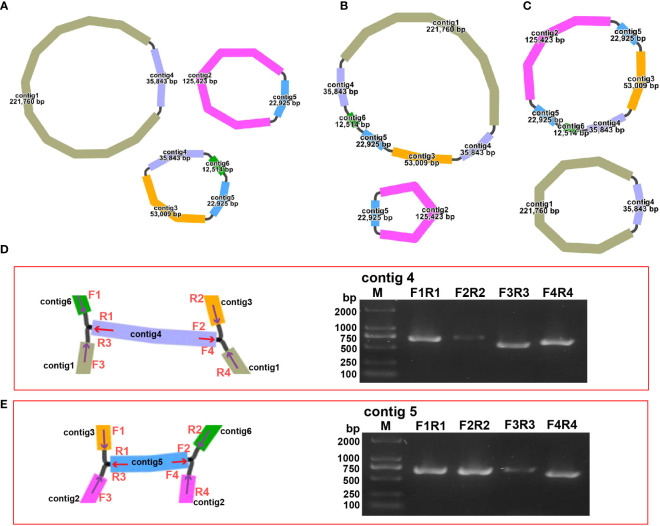
Recombination structure prediction and verification in *S. samarangense*. **(A)** Secondary conformation 1 (including 3 small circles) mediated by R1 and R2; **(B)** Secondary conformation 2 (including 2 small circles) mediated by R2; **(C)** Secondary conformation 3 (including 2 small circles) mediated by R1. **(D)** Verification of junction sequences in R1. **(E)** Verification of junction sequences in R2.

The structure of the plant mitogenome is dynamic owing to long repetitive sequences that can mediate genome recombination causing conformational changes (Gualberto and Newton, 2017; Kozik et al., 2019). It can be reasonably deduced that a single circular molecule is insufficient for the comprehensive display of the *S. samarangense* mitogenome structure. There may be multiple connections at branch nodes, and potential secondary conformations mediated by R1 and/or R2 (**Table 2**), as shown in **Figures 4A–C**. R1 and R2-mediated recombination was identified using a junction approach with validation using newly designed primers (**Supplementary Table 7**) and electrophoresis (**Figures 4D, E**). Detailed sequencing comparisons are shown in **Supplementary Figure 3**. In summary, the *S. samarangense* mitogenome had a variety of potential recombination conformations.

**Table 2:** List of two repeat sequences mediated recombination in the *S. samarangense* mitogenome.

Repeat Name	Repeat 1	Repeat 2
Identities (%)	100	100
Length (bp)	35,843	22,925
Position-1	201,357-237,200	125,423-148,348
Position-2	458,960-494,803	507,317-530,242
E-value	0	0
Type	Forward	Forward

### Chloroplast genome and sequence migration analysis

**Figure 5:**
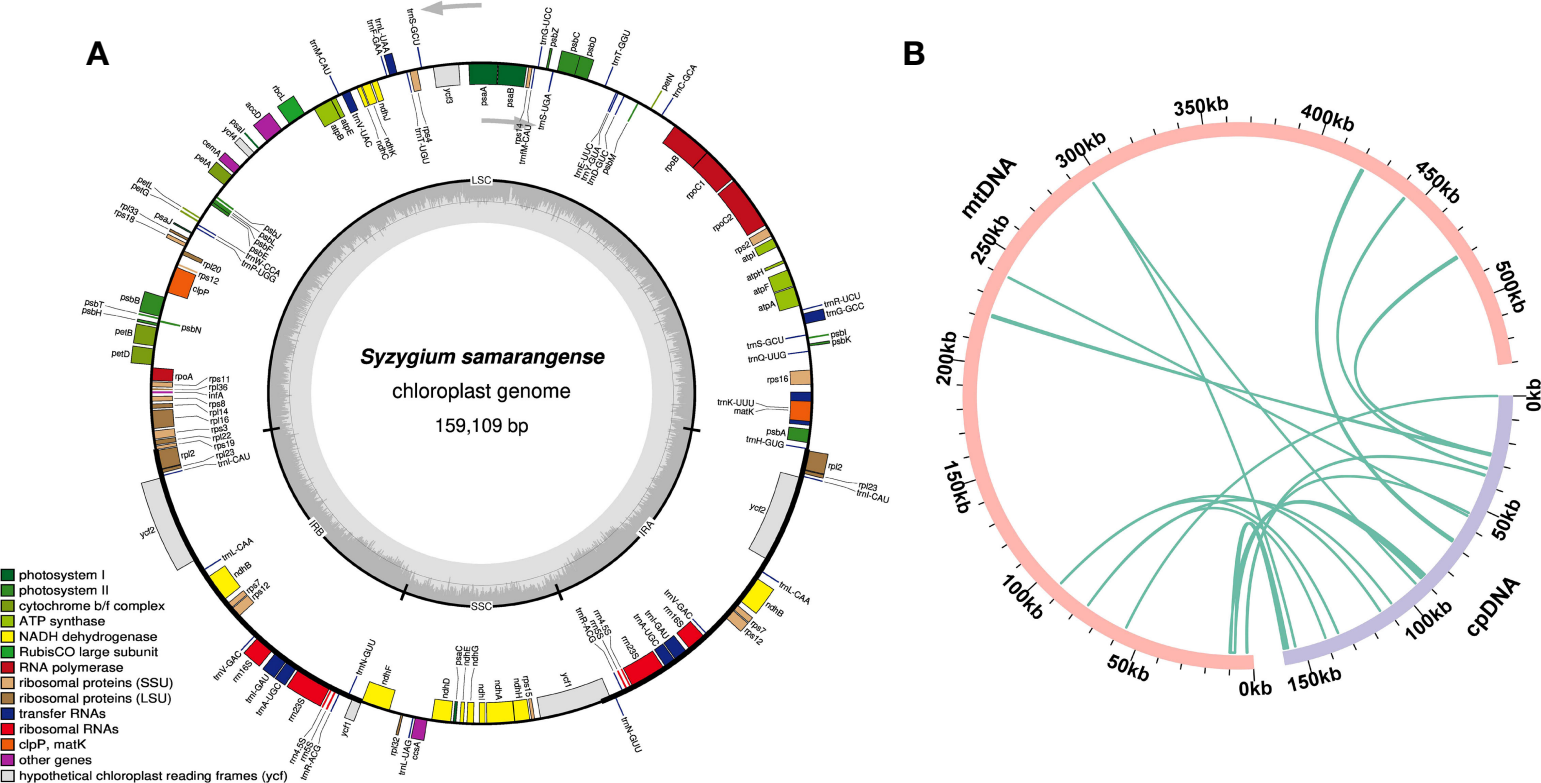
Chloroplast genome of *S. samarangense* **(A)** and sequence migration analysis between the chloroplast genome and mitochondrial genome **(B)**. Pink and light purple arcs represent the mitogenome and the chloroplast genome, respectively, and the dark green lines between the arcs correspond to the homologous fragments.

Sequence migration between the chloroplast and plant mitochondrial genomes has been prevalent throughout their long-term evolution (Prasad et al., 2022). The chloroplast genome of *S. samarangense* was assembled using the same sequencing data, with a size of 159,109 bp (**Figure 5A**). Thirteen homologous fragments of the chloroplast genome and mitochondrial genome were identified based on sequence similarity (**Figure 5B**, **Supplementary Table 8**), with a total length of 8,100 bp accounting for 1.53% of the total mitogenome length. Mitochondrial plastid sequence seven (MTPT7) was the longest (2,432 bp). Eighteen unique genes were identified after annotating these homologous sequences, of which ten were complete, including six PCGs (*psbJ*, *psbL*, *psbF*, *psbE*, *rps19*, and *rpl2*) and four tRNA genes (*trnD-GUC*, *trnH-GUG*, *trnM-CAU*, and *trnN-GUU*). The eight incomplete genes included *ndhC*, *psaD*, *rpl22*, *rpl23*, *rpoB*, *ycf2*, *trnI-GAU*, and *trnV-UAC*. However, these chloroplast genes transferred to the mitochondrial genome have undergone the varying degrees of sequence duplication, substitution, mutation, or loss during evolution, which have hindered their ability to perform their normal functions and caused them to evolve into pseudogenes (Mower et al., 2010; Mower et al., 2012).

### Phylogeny and collinearity analysis

**Figure 6:**
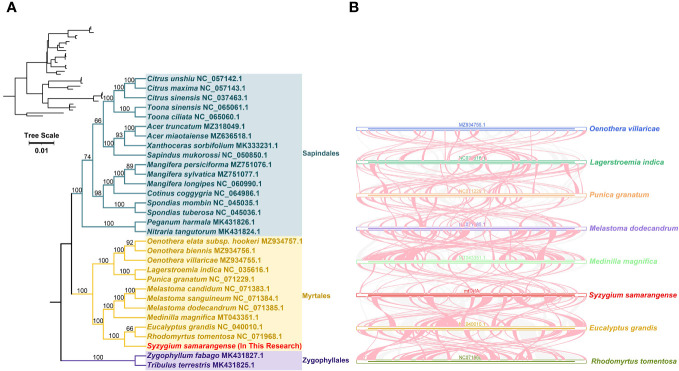
**(A)** Phylogenetic analysis of *S. samarangense* and related genera. Numbers at nodes show the bootstrap support values, and different colors indicate different Orders. **(B)** Collinearity analysis between *S. samarangense* and related genera. Blue, dark green, orange, purple, light green, red, yellow, and yellow-green lines represent *Oenothera villaricae*, *Lagerstroemia indica*, *Punica granatum*, *Melastoma dodecandrum*, *Medilla magnifica*, *Syzygium samarangense*, *Eucalyptus grandis*, and *Rhodomyrtus tomentosa*, respectively. Red curved areas indicate regions where inversions occur, and the gray areas indicate regions with high homology.

The loss or gain of PCGs commonly occurs during plant mitogenome evolution (Skippington et al., 2015; Wu et al., 2022). Only 23 out of 37 PCGs from the genomes of closely related species included in the phylogenetic analysis were shared: *atp1*, *atp4*, *atp6*, *atp8*, *ccmB*, *ccmC*, *ccmFC*, *ccmFN*, *cob*, *cox1*, *cox2*, *cox3*, *nad1*, *nad2*, *nad4*, *nad5*, *nad6*, *nad7*, *nad9*, *rpl5*, *rpl16*, *rps3*, and *sdh4*. The phylogenetic tree showed that *S. samarangense* was most closely related to *Eucalyptus grandis* and *Rhodomyrtus tomentosa* in the Myrtle family (**Figure 6A**). Moreover, the topology of the phylogeny of the above species (based on mitochondrial DNA) coincided with the latest classification of the Angiosperm Phylogeny Group.

A collinearity analysis of *S. samarangense* and seven Myrtales species (**Figure 6B**, **Supplementary Table 9**) revealed many homologous co-linear blocks, although the sizes of these co-linear blocks were generally short. Moreover, the co-linear block arrangement between the individual mitogenomes was inconsistent. It was hypothesized that extensive genomic reorganization occurred during the evolution of *S. samarangense* and closely related species. In addition, the identification of unique regions in species that lack homology with other species indicated species specificity in evolution.

### RNA editing events

**Figure 7:**
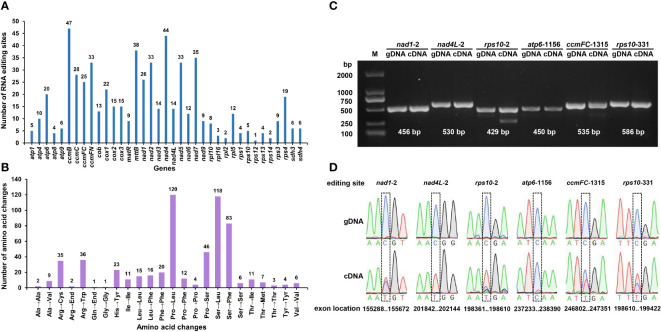
Predicted RNA editing sites **(A)** and corresponding amino acid changes **(B)** in *S. samarangense* mitogenome PCGs. PCR products **(C)** and sequence comparisons **(D)** for six specific editing sites.

RNA editing, one of the steps required for gene expression, is prevalent in higher plant mitochondria (Takenaka et al., 2013). RNA editing events were predicted for 37 PCGs of the *S. samarangense* mitogenome based on transcriptome data. They were observed in all of those genes, with a total of 591 RNA editing sites (**Figure 7A**), of which 473 had an editing frequency ≥ 0.80 (**Supplementary Table 10**). All were base C to U edits, and their RNA-seq mapping results were uploaded to the figshare platform (doi: 10.6084/m9.figshare.22650238). Among them, 47 RNA editing sites were identified in the *ccmB* gene, which had the highest number of edits among all mitochondrial genes. This was followed by the *nad4* gene, with 44 RNA editing sites. Furthermore, these editing sites were primarily observed in the second and first bases of the coding amino acids, and the number was 336 (56.85% of the total) and 187 (31.64%), respectively.

Most of the editing events resulted in non-synonymous codon changes, primarily involving the following three amino acid changes: Pro to Leu (No.120), Ser to Leu (No.118), and Ser to Phe (No.83) (**Figure 7B**). We predicted one ACG (Thr) to AUG (Met) editing event in *nad1*, *nad4L*, and *rps10*; one CAA (Gln) to UAA (End) in *atp6*; and one CGA (Arg) to UGA (End) in *ccmFC* and *rps10*. Further, we validated these six editing sites by PCR (**Figure 7C**) and Sanger sequencing (**Figure 7D**, **Supplementary file 1**). The editing frequency of the *atp6*-1156 editing site was low (the red line indicates base T in transcription). However, the specific effects of changes in the start and stop codons caused by RNA editing events in the *S. samarangense* mitogenome require in-depth studies.

## Discussion

A high-quality *S. samarangense* mitogenome with a length of 530,242 bp was assembled. The size of this genome varies slightly from that of other Myrtaceae species and differs significantly from those of *Fragaria* (275.2–351.2 Kb) (Fan et al., 2022), *Mangifera indica* (714.4–750.9 Kb) (Niu et al., 2022), and *Rhynchospora* (1.7–2.2 Mb) (Hofstatter et al., 2022). The 45.03% GC content in the *S. samarangense* mitogenome was not significantly different from those of the above species. This indicated that the GC content of the plant mitogenome was relatively conserved during evolution. *L. tulipifera* is an ancient legacy plant whose mitogenome evolved very slowly, retaining genes frequently lost in angiosperms and containing 41 PCGs (Richardson et al., 2013). At least four PCGs (*rps2*, *rps7*, *rps11*, and *rps19*) were lost in the *S. samarangense* genome. This indicated that a gene loss or transfer event occurred in this species, and ribosomal protein genes are more likely to be lost (Adams and Palmer, 2003). The coding region of the *S. samarangense* mitogenome only accounted for 7.99% of its full length with >90% non-coding regions; the gene distribution was very sparse, with highly conserved coding sequences. This proportion of the genome that is coding was similar to those in *L. tulipifera* (7.7%, excluding *cis*-spliced introns) (Richardson et al., 2013) and *Populus simonii* (8.25%) (Bi et al., 2022).

Repeats are identical or symmetrical DNA sequence fragments that frequently occur in plant genomes, occupy a large proportion of plant mitogenomes, and have important roles in genome size evolution, gene expression regulation, and responses to stress (Wendel et al., 2016). They are classified into tandem repeats and interspersed repeats according to whether they are adjacent or not. SSRs are a special kind of tandem repeat (generally not more than 6 bp) that are often used as genetic markers in plants (Khera et al., 2015). This study identified a total of 167 SSRs in the *S. samarangense* mitogenome, providing a large number of referenceable markers for germplasm diversity evaluation and species identification. Interspersed repeat sequences (also known as transposable elements) are a class of DNA sequences that can move their position on the genome, leading to gradual changes in functional genes and contributing to evolution (Bennetzen and Wang, 2014). They can regulate gene expression and affect plant phenotypic traits (Lisch, 2013), such as plant height and ear height in maize (Li et al., 2020), pigment formation in blood orange (Butelli et al., 2012), fruit shape in tomato (Xiao et al., 2008), and aluminum tolerance in wheat (Tovkach et al., 2013). In this study, 529 pairs of interspersed repeats were detected in the *S. samarangense* mitogenome; however, their effects on plant phenotypic traits require further in-depth study.

Repeat-mediated genome rearrangement is one of the main drivers of changes in mitogenome structure in plants, affecting gene organization and creating gene chimeras. This process affects plant phenotypes and evolution (Sanchez-Puerta et al., 2015; Gualberto and Newton, 2017). Large repetitive sequences over 1000 bp are more prone to recombination in the genome (Kozik et al., 2019). This study identified multiple potential conformations due to R1 (35,843 bp) and/or R2 (22,925 bp) repeat-mediated recombination in the *S. samarangense* mitogenome, although a single circle conformation containing information about the entire genome predominates (**Figures 1B**, **4A–C**). Therefore, rearrangement events in *S. samarangense* resulted in differentiation within the *Syzygium* genome.

Horizontal gene transfer is the transmission of genes or DNA fragments within cells or across species boundaries by asexual means; it is one of the main drivers of land plant evolution (Prasad et al., 2022). However, the introduced genes usually degenerate into pseudogenes (Mower et al., 2010). This study identified a total of 13 homologous fragments between the chloroplast genome and the *S. samarangense* mitogenome, accounting for 1.53% of the total mitogenome length. Among these homologous sequences, ten complete mitochondrial genes were identified, including six PCGs and four tRNA genes. The migration of gene sequences led to mitogenome expansion, which contributed to plant mitochondrial genetic diversity, although most of these migrating sequences lost their integrity during evolution (Alverson et al., 2011). Furthermore, only 23 PCGs were identical among the 31 closely related species in this study. This further suggested that gene gain/loss events occurred in closely related plants during evolution (Allen et al., 2007; Kubo and Newton, 2008).

RNA editing is a kind of nucleotide modification at the RNA level in higher plants and plays an essential role in plant adaptation and development. Changes in nonsynonymous codons caused by RNA editing may result in significant changes in gene function, especially with the creation of new start and stop codons (Ichinose and Sugita, 2017; Hao et al., 2021). RNA editing produces an ACG (Thr) to AUG (Met) editing events that can serve as the starting point for gene transcription (Quiñones et al., 1995; Zandueta-Criado and Bock, 2004). Similarly, RNA editing may produce stop codons that cause the premature termination of protein synthesis. Mitochondrial RNA editing truncated the *orf77* chimeric open reading frame associated with *S* cytoplasmic male sterility in maize, resulting in pollen abortion (Gallagher et al., 2002). Meanwhile, RNA editing changes from CAA (Gln) to UAA (End) at the *atp6*-1003 site ensured the normal synthesis of the polypeptide encoded by *atp6* in Yunnan purple rice maintenance lines, while sterile lines had no RNA editing at this site (Wei et al., 2010). Interestingly, this study identified six editing sites that could create start or stop codons: *nad1*-2 (Thr to Met), *nad4L*-2 (Thr to Met), *rps10*-2 (Thr to Met), *atp6*-1156 (Gln to End), *ccmFC*-1315 (Arg to End), and *rps10*-331 (Arg to End). Their specific roles in plant growth and development require in-depth exploration.

## Conclusions

In this study, we assembled the first complete mitogenome of *S. samarangense*. It was 530,242 bp in length, with a GC content of 45.03%, and encoded 61 unique genes. The many repetitive sequences and homologous segments of the chloroplast genome in the mitogenome of *S. samarangense* suggests that it may underwent multiple genomic recombination events during evolution. Moreover, two forward repeats were involved in mediating recombination. Therefore, multiple secondary conformations may exist in addition to the master circular structure. A total of 591 RNA editing sites were identified in the PCGs, among which six sites create start or stop codons and should be the focus of future studies. The newly obtained mitogenome can be used as a reference genome for analyses of other *Syzygium* species and provides important information for the molecular breeding of *S. samarangense*.

## Data availability statement

The datasets presented in this study can be found in online repositories. The names of the repository/repositories and accession number(s) can be found in the article/**Supplementary Material**. The mitogenome sequence supporting the conclusions of this article is available in GenBank (https://www.ncbi.nlm.nih.gov/) under accession number: OQ701348. The chloroplast genome sequence information has been uploaded to NCBI (accession number: OR766332). The Illumina, Nanopore, and transcriptome data of Syzygium samarangense have been deposited to the Sequence Read Archive repository under SRR26670650, SRR26670651, and SRR24058007, respectively.

## Author contributions

GL: Conceptualization, Data curation, Formal analysis, Methodology, Software, Validation, Writing – original draft. QL: Funding acquisition, Project administration, Resources, Supervision, Visualization, Writing – review & editing.
