# Supplementary material for: Complete mitochondrial genome of Syzygium samarangense reveals genomic recombination, gene transfer, and RNA editing events
Source: Front Plant Sci. 2024 Jan 9;14:1301164. doi: 10.3389/fpls.2023.1301164 (PMC10803518; doi:10.3389/fpls.2023.1301164)
Supplement: Supplementary file 1 [file DataSheet_1.zip › Supplementary file 1/atp6-stop.pdf]

10 20 30 40 50 60

ATGTTTGAAAAATTTCCACGCCTCTTTTTATTTGATGAAAATAGTCTGAATTCCAGTGCTACATCTT

Syzygium_samarangense_contig1(1>1158) → ATGTTTGAAAAATTTCCACGCCTCTTTTTATTTGATGAAAATAGTCTGAATTCCAGTGCTACATCTT

70 80 90 100 110 120 130

CTAGAACTCCGAGTCAATCCACGACGACTATTACCGATTATAGTCTTCAATCGTCCGATACTCAAGG

Syzygium_samarangense_contig1(1>1158) → CTAGAACTCCGAGTCAATCCACGACGACTATTACCGATTATAGTCTTCAATCGTCCGATACTCAAGG

140 150 160 170 180 190 200

TTCTTATGATGGTATTTTTGAGGATCATCCAGGTCTTAACCCTTCCAGTGAACGTATAGTAGAGCTT

Syzygium_samarangense_contig1(1>1158) → TTCTTATGATGGTATTTTTGAGGATCATCCAGGTCTTAACCCTTCCAGTGAACGTATAGTAGAGCTT

210 220 230 240 250 260

CAATCTGATATACGCGAGAAATTGGAGGAGCTTCTGATTAACAAGAGTCCCGACGAAGCTATGGTTG

Syzygium_samarangense_contig1(1>1158) → CAATCTGATATACGCGAGAAATTGGAGGAGCTTCTGATTAACAAGAGTCCCGACGAAGCTATGGTTG

270 280 290 300 310 320 330

CGGCCGAAGCTTTACATGGGGAAAGCGACAATATCCCTTTTATGGAGTTCATTTTGAATGATTTGAA

Syzygium_samarangense_contig1(1>1158) → CGGCCGAAGCTTTACATGGGGAAAGCGACAATATCCCTTTTATGGAGTTCATTTTGAATGATTTGAA

340 350 360 370 380 390 400

CACAAACGGAGTAGAAAGTGAGTCCTATAAAGATGCGCTGGCAGCACTAGTGAAGATGGTCCCCAGC

Syzygium_samarangense_contig1(1>1158) → CACAAACGGAGTAGAAAGTGAGTCCTATAAAGATGCGCTGGCAGCACTAGTGAAGATGGTCCCCAGC

410 420 430 440 450 460

CCACTTGAACAATTTGAAATCATCCCATTTTTTCCTATGAAGATAGGAGACTTGTATTTCTCATTCA

Syzygium_samarangense_contig1(1>1158) → CCACTTGAACAATTTGAAATCATCCCATTTTTTCCTATGAAGATAGGAGACTTGTATTTCTCATTCA

470 480 490 500 510 520 530

CAAATCCATCTTTGTTTATGCTGCTAACTCTCAGTTTGGTCCTACTTCTGCTTTATTTTGTTACTAA

Syzygium_samarangense_contig1(1>1158) → CAAATCCATCTTTGTTTATGCTGCTAACTCTCAGTTTGGTCCTACTTCTGCTTTATTTTGTTACTAA

540 550 560 570 580 590 600

AAAGGGAGGAGGAAACTCAGTACCAAATGCTTGGCAATCCTTGGTAGAGCTTATTTATGATTTCGTG

Syzygium_samarangense_contig1(1>1158) → AAAGGGAGGAGGAAACTCAGTACCAAATGCTTGGCAATCCTTGGTAGAGCTTATTTATGATTTCGTG

610 620 630 640 650 660 670

CTAAACCTGGTAAACGAACAAATAAGTGGTCTTTCCGGAAATGTGAAACAAAAGTTTTTCCCTTGCA

Syzygium_samarangense_contig1(1>1158) → CTAAACCTGGTAAACGAACAAATAAGTGGTCTTTCCGGAAATGTGAAACAAAAGTTTTTCCCTTGCA

680 690 700 710 720 730

TATTGGTCACTTTTACTTTTTCGTTATTTCGTAATCCCCAGGGTATGATACCTTATAGCTTCACAGT

Syzygium_samarangense_contig1(1>1158) → TATTGGTCACTTTTACTTTTTCGTTATTTCGTAATCCCCAGGGTATGATACCTTATAGCTTCACAGT

740 750 760 770 780 790 800

TACAAGTCATTTTCTCATTACTTTGGGTCTCTCATTTTCTATTTTTATAGGCATTACTATAGTGGGA

Syzygium_samarangense_contig1(1>1158) → TACAAGTCATTTTCTCATTACTTTGGGTCTCTCATTTTCTATTTTTATAGGCATTACTATAGTGGGA

810 820 830 840 850 860 870

TTTCAAAGAAATGGGCTTCATTTTTTAAGCTTCTTATTACCCGCAGGAGTCCCACTGCCGTTAGCAC

Syzygium_samarangense_contig1(1>1158) → TTTCAAAGAAATGGGCTTCATTTTTTAAGCTTCTTATTACCCGCAGGAGTCCCACTGCCGTTAGCAC

880 890 900 910 920 930

CTTTTTTAGTACTCCTTGAGC-T-A-A-TCCCTCATTGTTTTCGCGCATTAAGCTCAGGAATACGTT

Syzygium_samarangense_contig1(1>1158) CTTTTTTAGTACTCCTTGAGC-T-A-A-TCCCTCATTGTTTTCGCGCATTAAGCTCAGGAATACGTT

gDNA-atp6-stop.ab1(22>291)

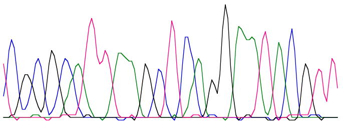

CGCGCATTAAGCTCAGGAATACGTT

cDNA-atp6-stop.ab1(24>293)

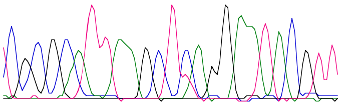

CGCGCATTAAGCTCAGGAATACGTT

940 950 960 970 980 990 1000

TATTTGCTAATATGATGGCCGGTCATAGTCCAGTAAAGATTTTAAGTGGGTCCGCTTGGACTATGCT

Syzygium_samarangense_contig1(1>1158) TATTTGCTAATATGATGGCCGGTCATAGTCCAGTAAAGATTTTAAGTGGGTCCGCTTGGACTATGCT

gDNA-atp6-stop.ab1(22>291)

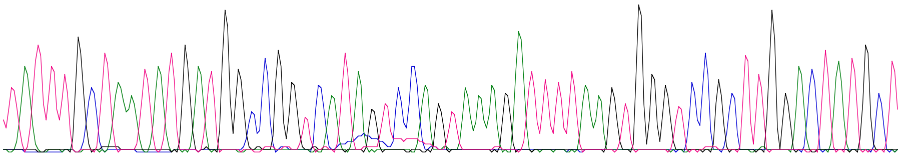

TATTTGCTAATATGATGGCCGGTCATAGTCCAGTAAAGATTTTAAGTGGGTCCGCTTGGACTATGCT

cDNA-atp6-stop.ab1(24>293)

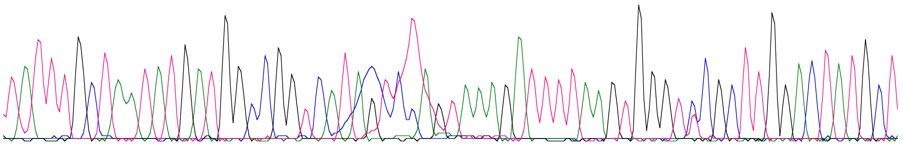

TATTTGCTAATATGATGGCCGGTCATAGTCCAGTAAAGATTTTAAGTGGGTCCGCTTGGACTATGCT

1010 1020 1030 1040 1050 1060 1070

ATGTATGAATGATCTTTTCTATTTCATAGGGGATCTTGGTCCTTTATTTATAGTTCTTGCATTAACC

Syzygium_samarangense_contig1(1>1158) ATGTATGAATGATCTTTTCTATTTCATAGGGGATCTTGGTCCTTTATTTATAGTTCTTGCATTAACC

gDNA-atp6-stop.ab1(22>291)

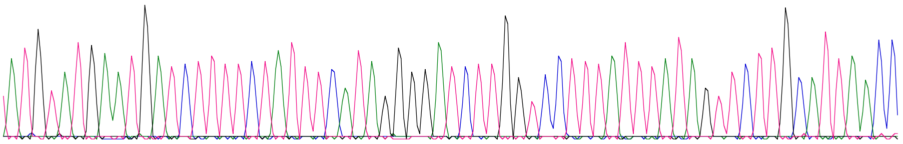

ATGTATGAATGATCTTTTCTATTTCATAGGGGATCTTGGTCCTTTATTTATAGTTCTTGCATTAACC

cDNA-atp6-stop.ab1(24>293)

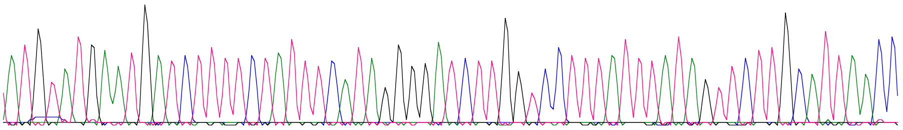

ATGTATGAATGATCTTTTCTATTTCATAGGGGATCTTGGTCCTTTATTTATAGTTCTTGCATTAACC

1080 1090 1100 1110 1120 1130

GGTCCGGAATTAGGTGTAGCTATATCACAAGCTCATGTTTCTACGATCTCAATCTGTATTTACTTGA

Syzygium_samarangense_contig1(1>1158) GGTCCGGAATTAGGTGTAGCTATATCACAAGCTCATGTTTCTACGATCTCAATCTGTATTTACTTGA

gDNA-atp6-stop.ab1(22>291)

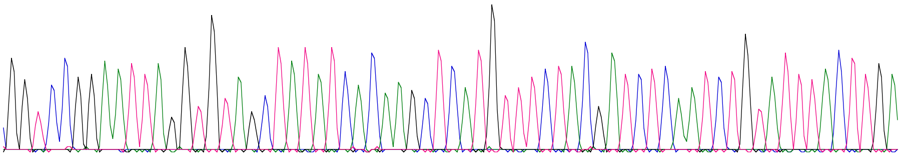

GGTCCGGAATTAGGTGTAGCTATATCACAAGCTCATGTTTCTACGATCTCAATCTGTATTTACTTGA

cDNA-atp6-stop.ab1(24>293)

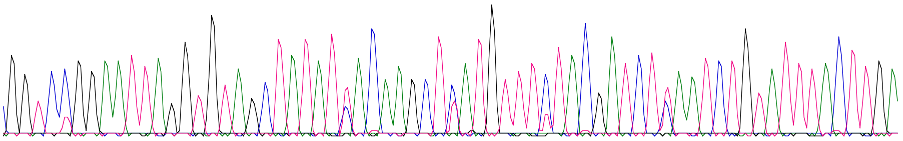

GGTCCGGAATTAGGTGTAGCTATATCACAAGCTCATGTTTCTACGATCTCAATCTGTATTTACTTGA

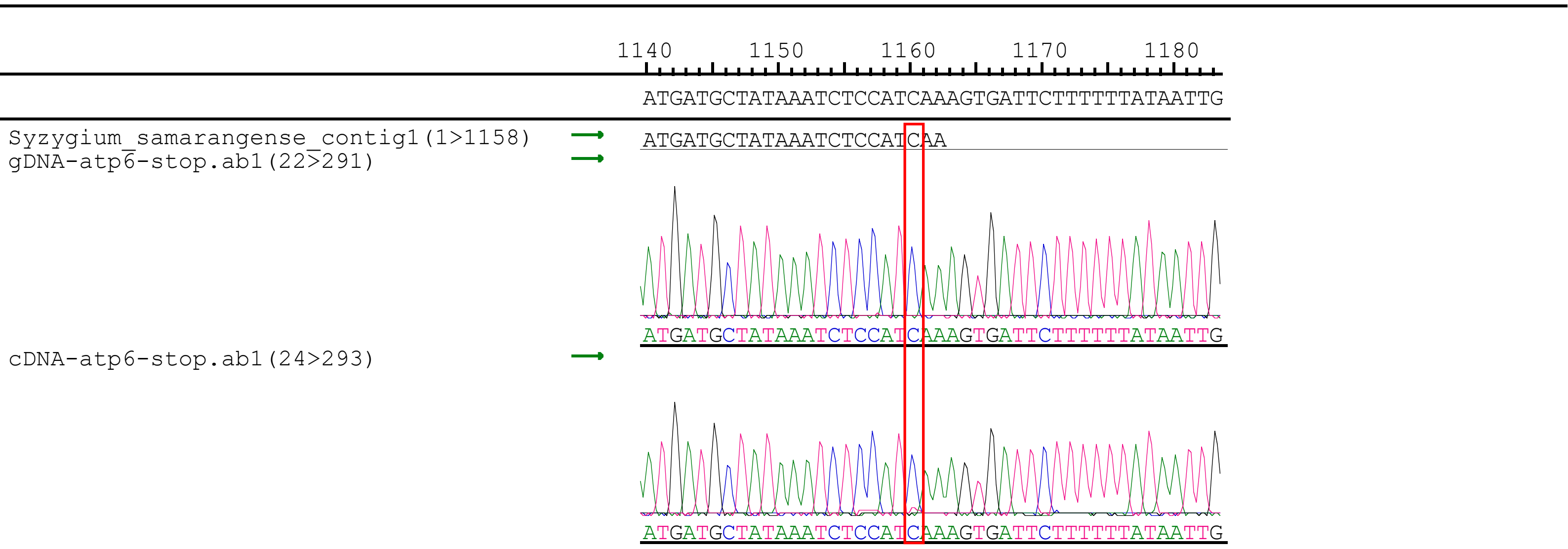
1140
1150
1160
1170
1180
ATGATGCTATAAATCTCCATCAAAGTGATTCTTTTTTATAATTG
Syzygium_samarangense_contig1(1>1158)
ATGATGCTATAAATCTCCATCAA
gDNA-atp6-stop.ab1(22>291)
ATGATGCTATAAATCTCCATCAAAGTGATTCTTTTTTATAATTG
cDNA-atp6-stop.ab1(24>293)
ATGATGCTATAAATCTCCATCAAAGTGATTCTTTTTTATAATTG
