# Supplementary material for: Complete mitochondrial genome of Syzygium samarangense reveals genomic recombination, gene transfer, and RNA editing events
Source: Front Plant Sci. 2024 Jan 9;14:1301164. doi: 10.3389/fpls.2023.1301164 (PMC10803518; doi:10.3389/fpls.2023.1301164)

10 20 30 40 50 60

ATGGTCCAACTACATAACTTTTTCTTTTTCATTACTTCCATGGTCGTGCCTCGTGGCACGGCAGCAC

Syzygium_samarangense_contig1(1>1317) → ATGGTCCAACTACATAACTTTTTCTTTTTCATTACTTCCATGGTCGTGCCTCGTGGCACGGCAGCAC

70 80 90 100 110 120 130

CCGTACTATTGAAATGGTTCGTCAGTAGAGATGTTCCCACAGGTGCCCCTTCTTCCAATGGAACTAT

Syzygium_samarangense_contig1(1>1317) → CCGTACTATTGAAATGGTTCGTCAGTAGAGATGTTCCCACAGGTGCCCCTTCTTCCAATGGAACTAT

140 150 160 170 180 190 200

AATTCCTATTCCTATCCCTTCATTCCCTCTTTTGGTCTATCTACATTCCAGGAAATTCATACGCTCC

Syzygium_samarangense_contig1(1>1317) → AATTCCTATTCCTATCCCTTCATTCCCTCTTTTGGTCTATCTACATTCCAGGAAATTCATACGCTCC

210 220 230 240 250 260

ATGGACGGAGCAAAAAGTGGAGTCTTGGTCAGAGCAAGCCACCCTATTCTATTACCAGACATAATTG

Syzygium_samarangense_contig1(1>1317) → ATGGACGGAGCAAAAAGTGGAGTCTTGGTCAGAGCAAGCCACCCTATTCTATTACCAGACATAATTG

270 280 290 300 310 320 330

GGAGAAGCTCATCCGAAACTAGAGCTAGAAAGGCCTCATTTCGTTTCGTTCCCGTTTTTATTATCCT

Syzygium_samarangense_contig1(1>1317) → GGAGAAGCTCATCCGAAACTAGAGCTAGAAAGGCCTCATTTCGTTTCGTTCCCGTTTTTATTATCCT

340 350 360 370 380 390 400

TCTTCTCGAATCCAAGGGGGACTTATCATATTTAGAATCTTTCTGCGGTGTGCTCCGTTTACTATTC

Syzygium_samarangense_contig1(1>1317) → TCTTCTCGAATCCAAGGGGGACTTATCATATTTAGAATCTTTCTGCGGTGTGCTCCGTTTACTATTC

410 420 430 440 450 460

TTTCGTACTCTCTTCTCTTTACCACGCGATAGGTCAGCGAAGCGTGAGCGGGCGCGGAGAAGGAAAG

Syzygium_samarangense_contig1(1>1317) → TTTCGTACTCTCTTCTCTTTACCACGCGATAGGTCAGCGAAGCGTGAGCGGGCGCGGAGAAGGAAAG

470 480 490 500 510 520 530

GCCAAAGACTTCGGCCTAAGGGAAATGAGCAACGACGAAATGACAAAATGAGGTGCTCCGGGCACTC

Syzygium_samarangense_contig1(1>1317) → GCCAAAGACTTCGGCCTAAGGGAAATGAGCAACGACGAAATGACAAAATGAGGTGCTCCGGGCACTC

540 550 560 570 580 590 600

CCATTTAGAAAGAAGTTTAGAAGGTTTTGGGCCTGTAGCTTTTCCCGTCCCCCCTTCATCGGGTGGT

Syzygium_samarangense_contig1(1>1317) → CCATTTAGAAAGAAGTTTAGAAGGTTTTGGGCCTGTAGCTTTTCCCGTCCCCCCTTCATCGGGTGGT

610 620 630 640 650 660 670

GCTTGTGTGGGGGGTGTGCCACCAGAAATCGGGCTTGAAGCTCTCGCCTTACCAACGAGCCGACAGC

Syzygium_samarangense_contig1(1>1317) → GCTTGTGTGGGGGGTGTGCCACCAGAAATCGGGCTTGAAGCTCTCGCCTTACCAACGAGCCGACAGC

680 690 700 710 720 730

TGATGGCTGTTGGTCACGACTACTACCAAAAAGCTCCAATGAAGATGAATATTTCACATGTAGGAGT

Syzygium_samarangense_contig1(1>1317) → TGATGGCTGTTGGTCACGACTACTACCAAAAAGCTCCAATGAAGATGAATATTTCACATGTAGGAGT

740 750 760 770 780 790 800

GTGCATCTCTATGTTGGGTGTTCTTCTGTCTAACACAAAGAAGATAGAGTTCACTCAACGATTGCCT

Syzygium_samarangense_contig1(1>1317) → GTGCATCTCTATGTTGGGTGTTCTTCTGTCTAACACAAAGAAGATAGAGTTCACTCAACGATTGCCT

810 820 830 840 850 860 870

TTGGGTTCCGAACTCCATATGGGGAAGGAGCGTTGTTGTTTGCGAGGTCTCGATCATTTACATGGGC

Syzygium_samarangense_contig1(1>1317) → TTGGGTTCCGAACTCCATATGGGGAAGGAGCGTTGTTGTTTGCGAGGTCTCGATCATTTACATGGGC

880 890 900 910 920 930

CCACTTCTCATTCCATTTGTGGGAATTTGATGATCTATAAACCGTCCCTAAGGAACGATCGGCTCAT

Syzygium_samarangense_contig1(1>1317) → CCACTTCTCATTCCATTTGTGGGAATTTGATGATCTATAAACCGTCCCTAAGGAACGATCGGCTCAT

940 950 960 970 980 990 1000

GTTTGAGCATGATGAATCACTTCGTGCCGACCTGTTGCCAATAAACTTTGCGGCCTCATATGAGAAT

Syzygium_samarangense_contig1(1>1317) → GTTTGAGCATGATGAATCACTTCGTGCCGACCTGTTGCCAATAAACTTTGCGGCCTCATATGAGAAT

1010 1020 1030 1040 1050 1060 1070

GGAAAACTGGAGCATTTTCTGCATCGGTGGATGAAGAATCGCAAACATAAGAATTTCTGGTTGACCA

Syzygium_samarangense_contig1(1>1317) → GGAAAACTGGAGCATTTTCTGCATCGGTGGATGAAGAATCGCAAACATAAGAATTTCTGGTTGACCA

1080 1090 1100 1110 1120 1130

TGTTCCCAGAAAAAAGATACTTTA-GAGAAACGACGAGCACGACTGAAGTGGCTATACATACAAATC

Syzygium_samarangense_contig1(1>1317) → TGTTCCCAGAAAAAAGATACTTTA-GAGAAACGACGAGCACGACTGAAGTGGCTATACATACAAATC

1140 1150 1160 1170 1180 1190 1200

CATTTACGGATCTATATGCTTCGATTGGAACTGGAAGTTCCAGAACAGGCGGCTGGTATACCACCAT

Syzygium_samarangense_contig1(1>1317) → CATTTACGGATCTATATGCTTCGATTGGAACTGGAAGTTCCAGAACAGGCGGCTGGTATACCACCAT

gDNA-ccmFC-stop.ab1(81>277) →

cDNA-ccmFC-stop.ab1(80>276) →

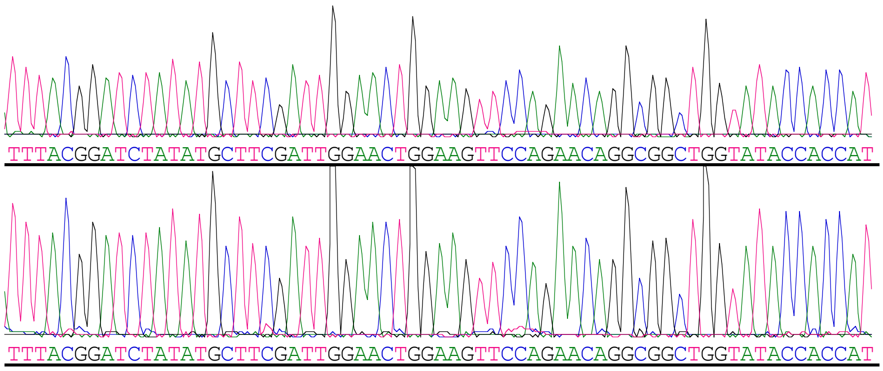

1210 1220 1230 1240 1250 1260 1270

AATGAAACTGCCTTTTCTTTTTTTTATTTGGATAGGATTTATGTTGGCTTCGTCGGGAGGCTCCCGT

Syzygium_samarangense_contig1(1>1317) → AATGAAACTGCCTTTTCTTTTTTTTATTTGGATAGGATTTATGTTGGCTTCGTCGGGAGGCTCCCGT

gDNA-ccmFC-stop.ab1(81>277) →

cDNA-ccmFC-stop.ab1(80>276) →

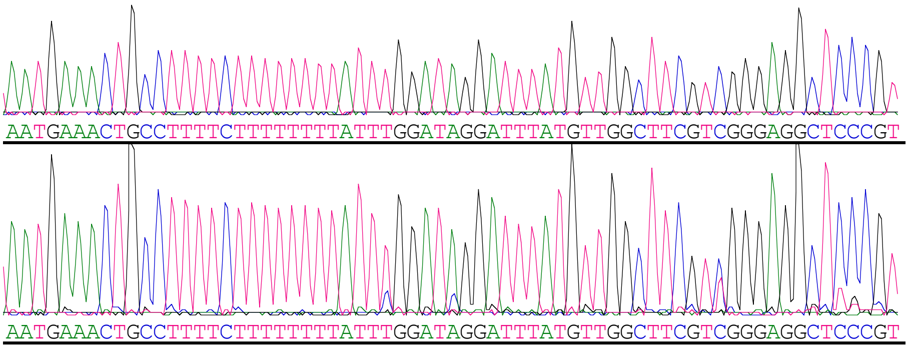

1280 1290 1300 1310 1320 1330

AGTTTGTTACATCAGCTCCAAAAGGATAAGTTGCGTTGGAATCGAGAAAGTTTCGTGGAGTTCAT

Syzygium_samarangense_contig1(1>1317) → AGTTTGTTACATCAGCTCCAAAAGGATAAGTTGCGTTGGAATCGA

gDNA-ccmFC-stop.ab1(81>277) →

cDNA-ccmFC-stop.ab1(80>276) →

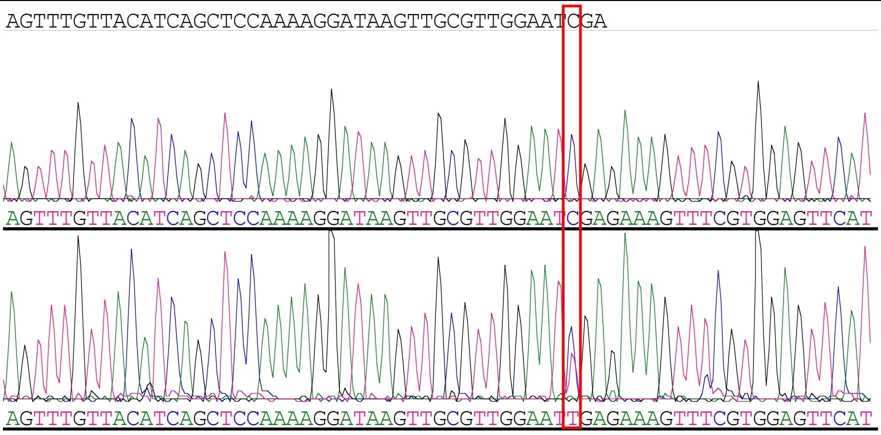

Supplement: Supplementary file 1 [file DataSheet_1.zip › Supplementary file 1/ccmFC-stop.pdf]
