# Supplementary material for: Complete mitochondrial genome of Syzygium samarangense reveals genomic recombination, gene transfer, and RNA editing events
Source: Front Plant Sci. 2024 Jan 9;14:1301164. doi: 10.3389/fpls.2023.1301164 (PMC10803518; doi:10.3389/fpls.2023.1301164)
Supplement: Supplementary file 1 [file DataSheet_1.zip › Datasheet 1.docx]

# Supplementary Figures


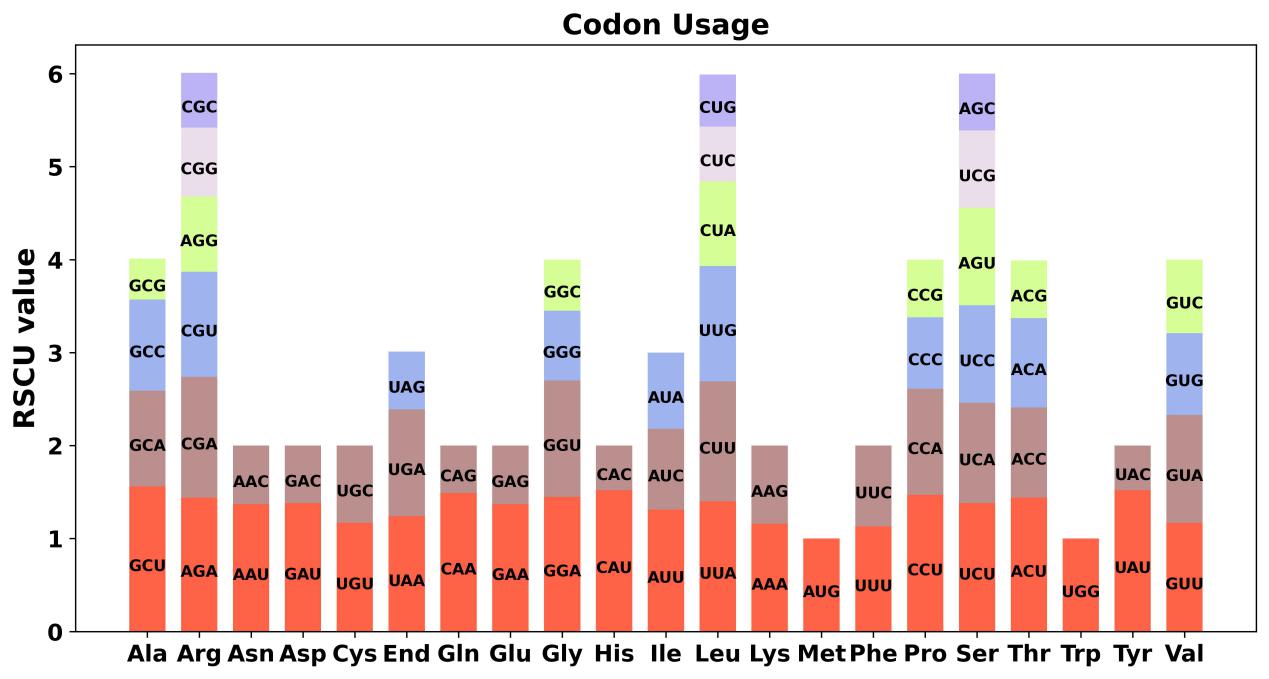


**Figure S1**. Codon usage preference of protein-coding genes in *Syzygium samarangense*


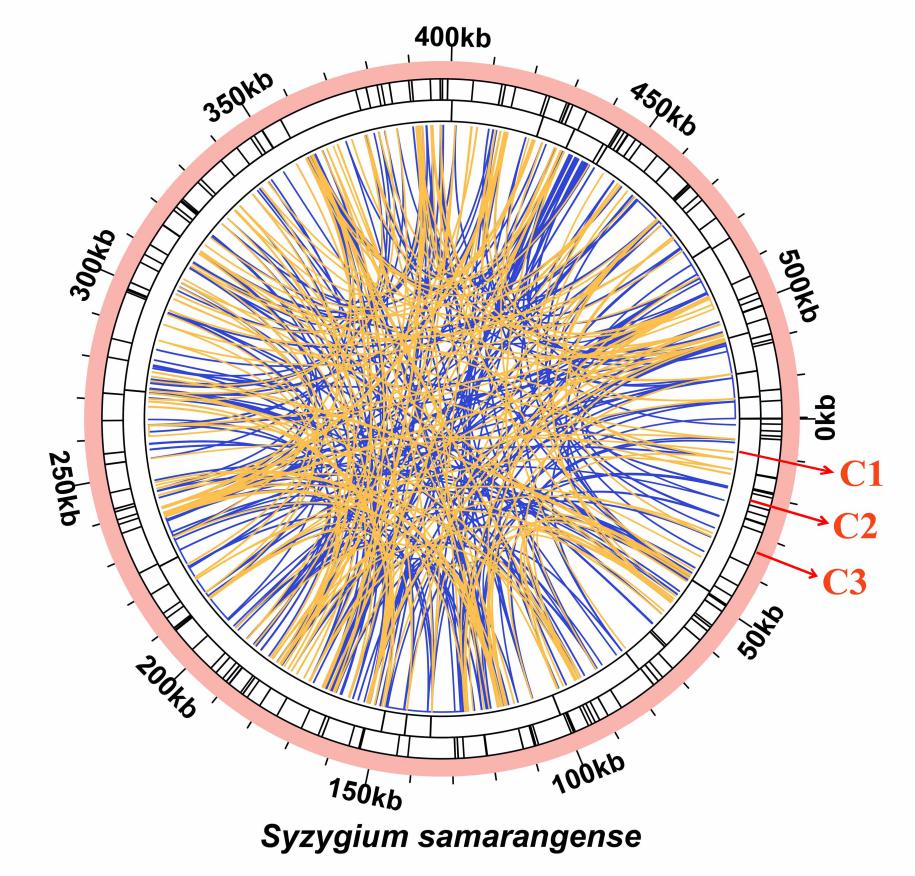


**Figure S2**. Repeat sequences of the *Syzygium samarangense* mitogenome

The color line on the C1 circle connects two repeated dispersed repeats. The yellow and blue lines represent palindromic repeats and forward repeats, respectively. The black line on the C2 circle represent tandem repeats. The black line on the C3 circle simple sequence repeats.


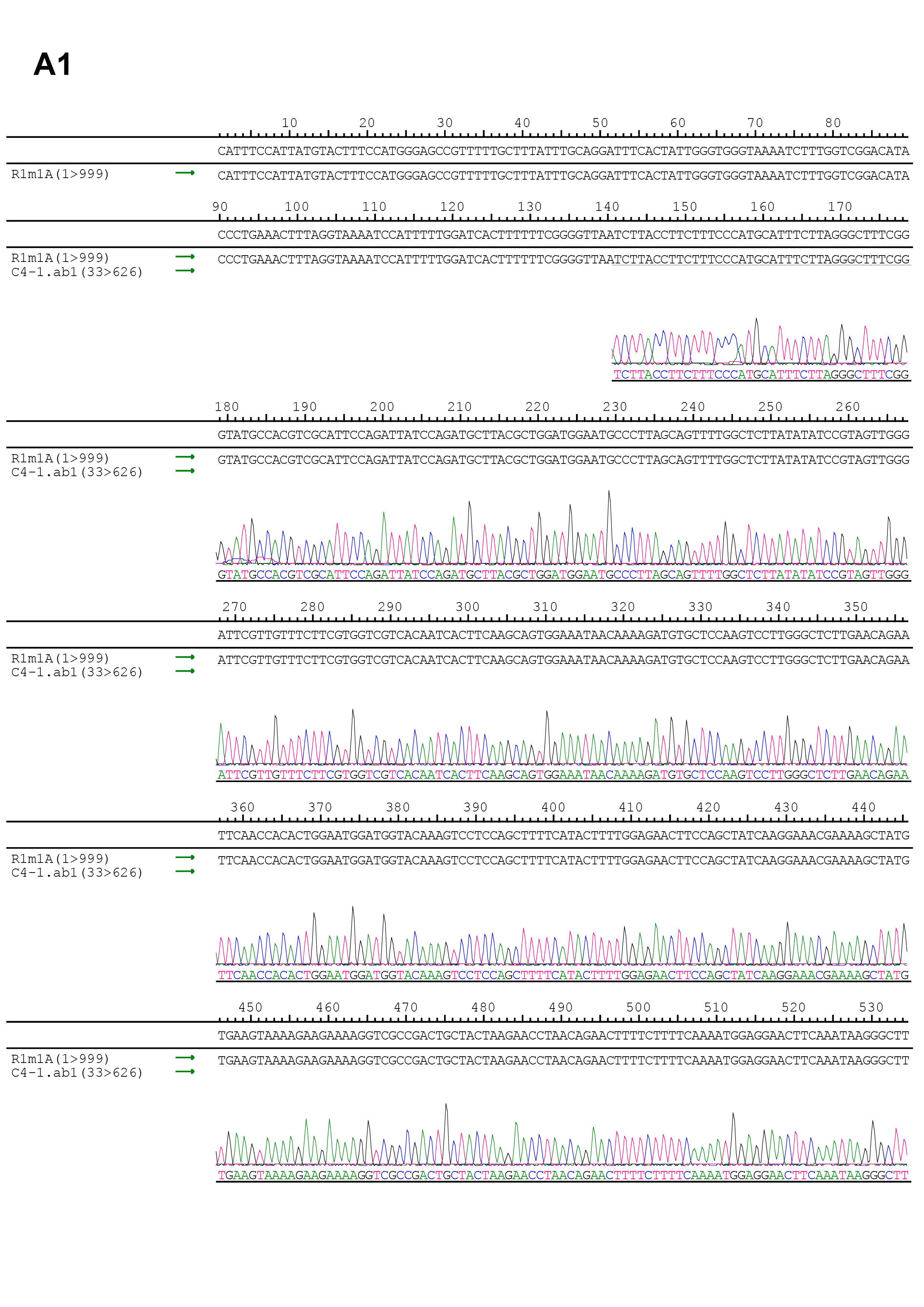


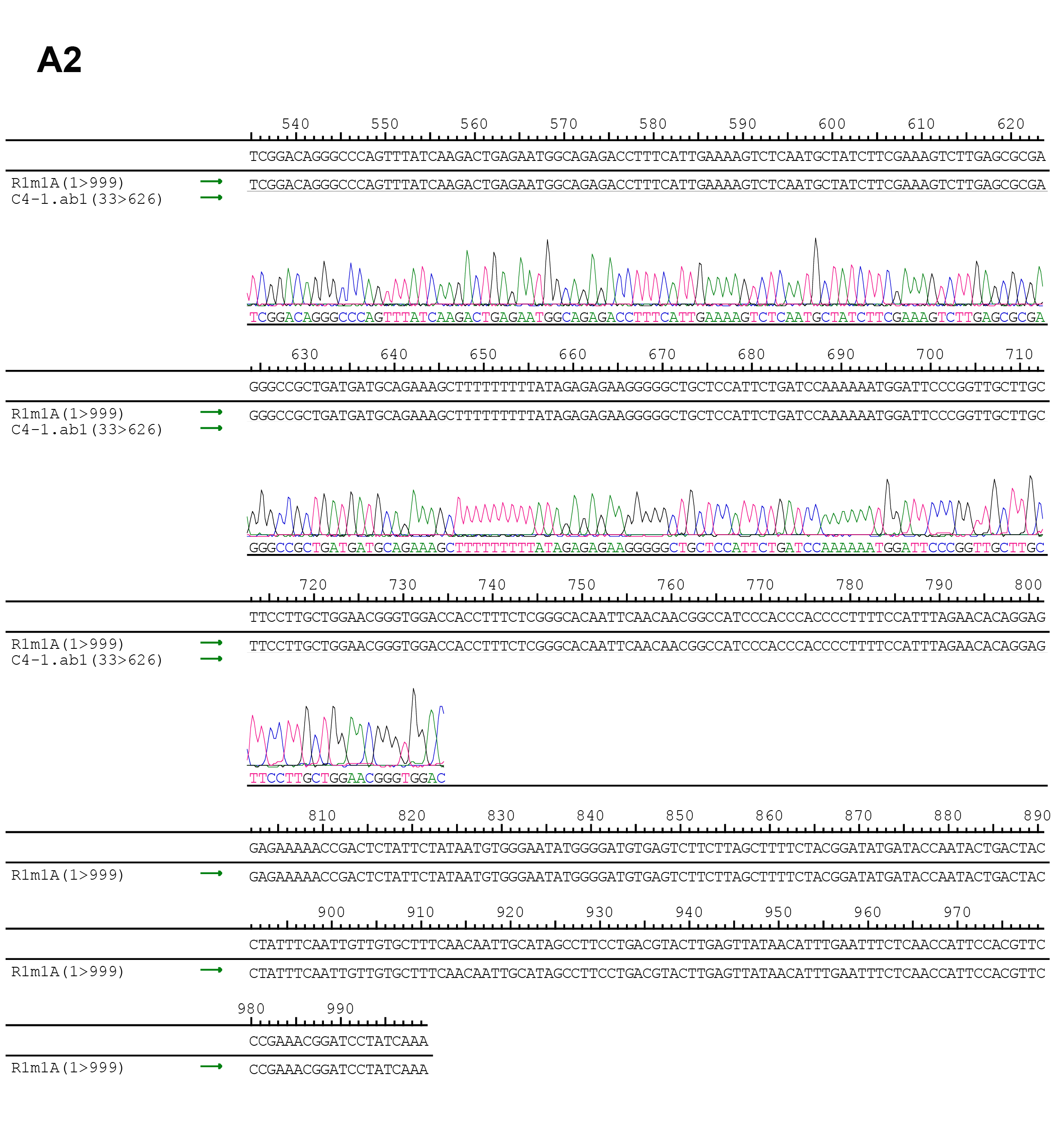


**Figure S3**. Boundary verification of repeat-mediated recombination (A: C4-F1)


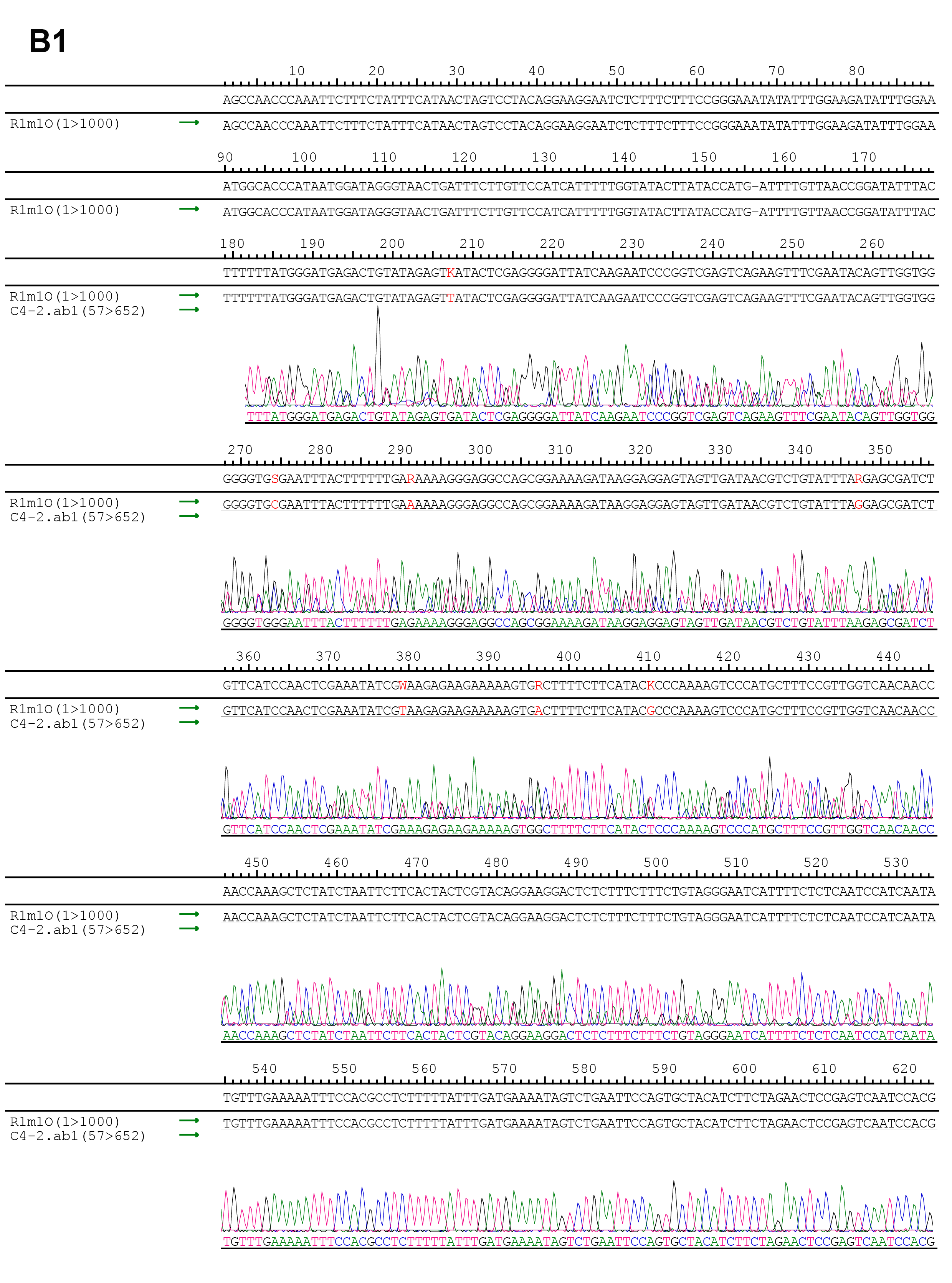


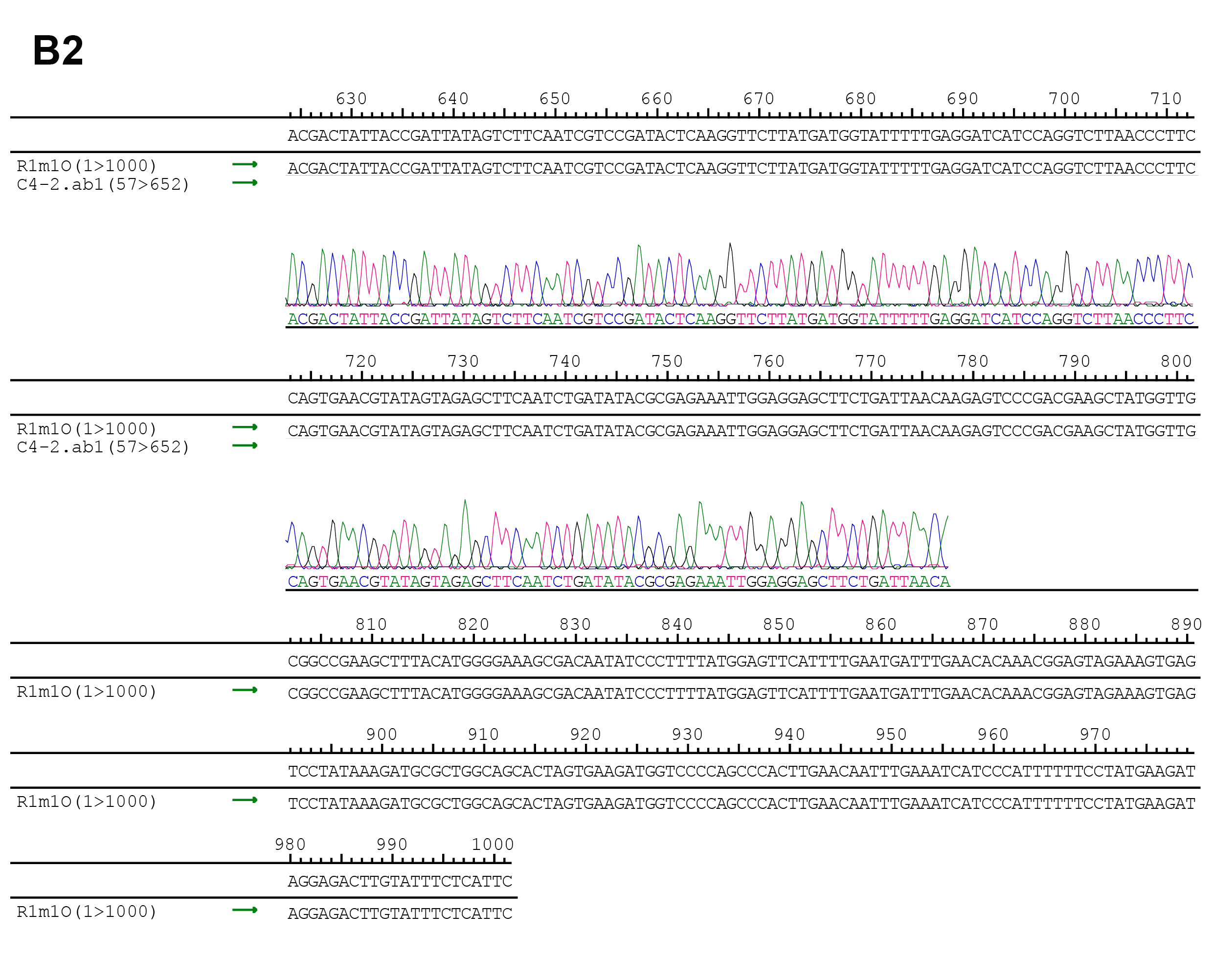


**Figure S3**. Boundary verification of repeat-mediated recombination (B: C4-R1)


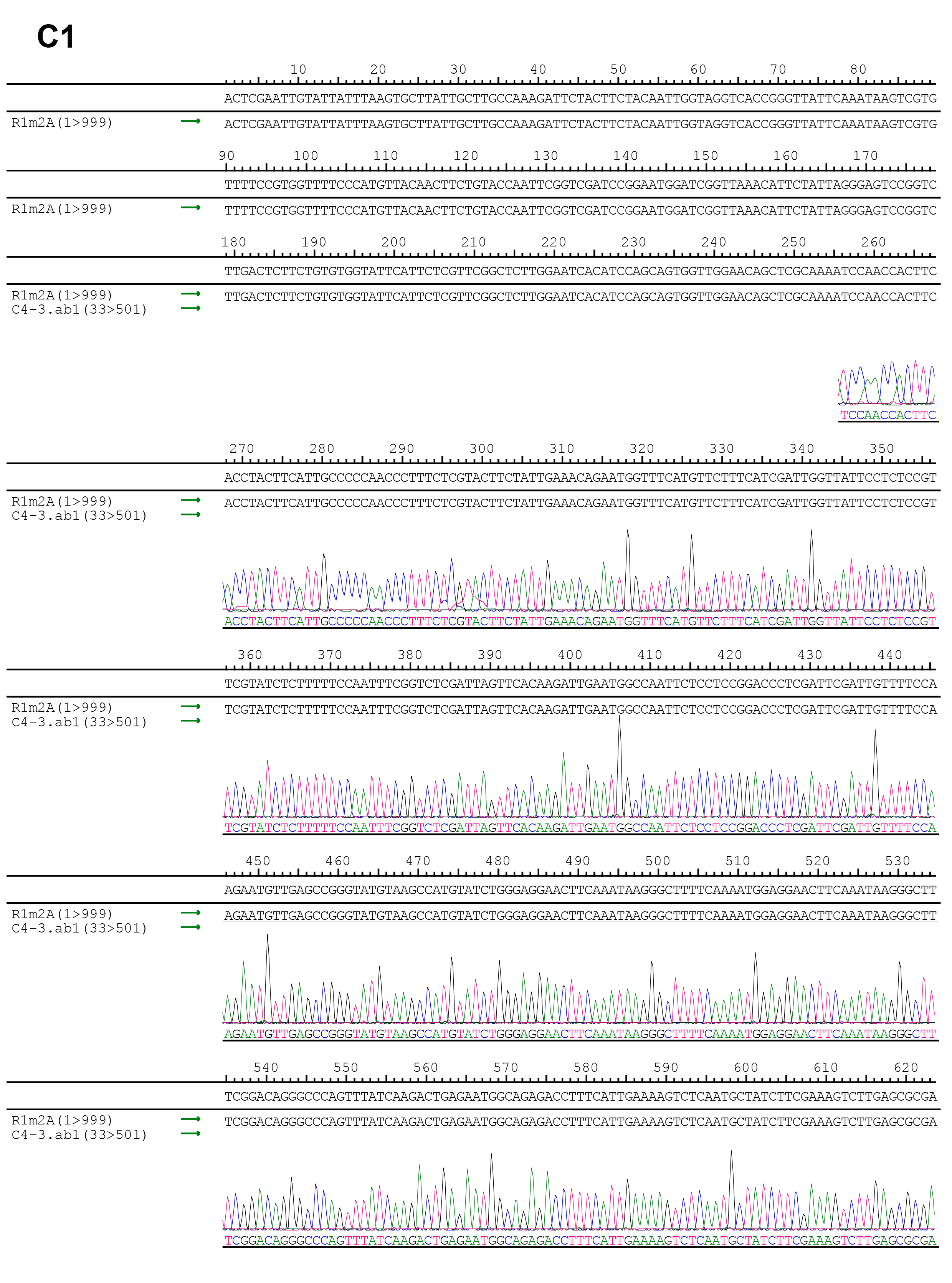


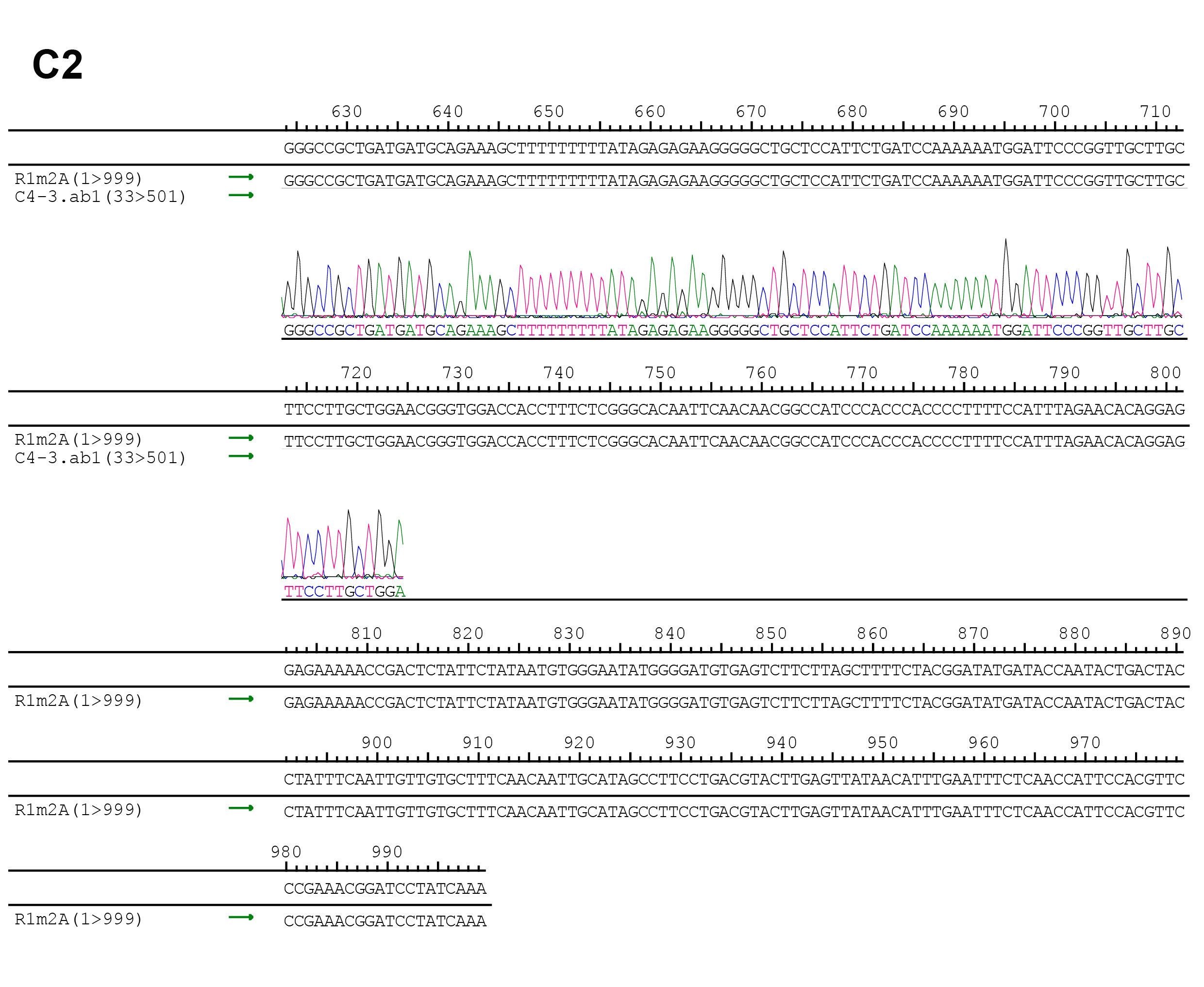


**Figure S3**. Boundary verification of repeat-mediated recombination (C: C4-F2)


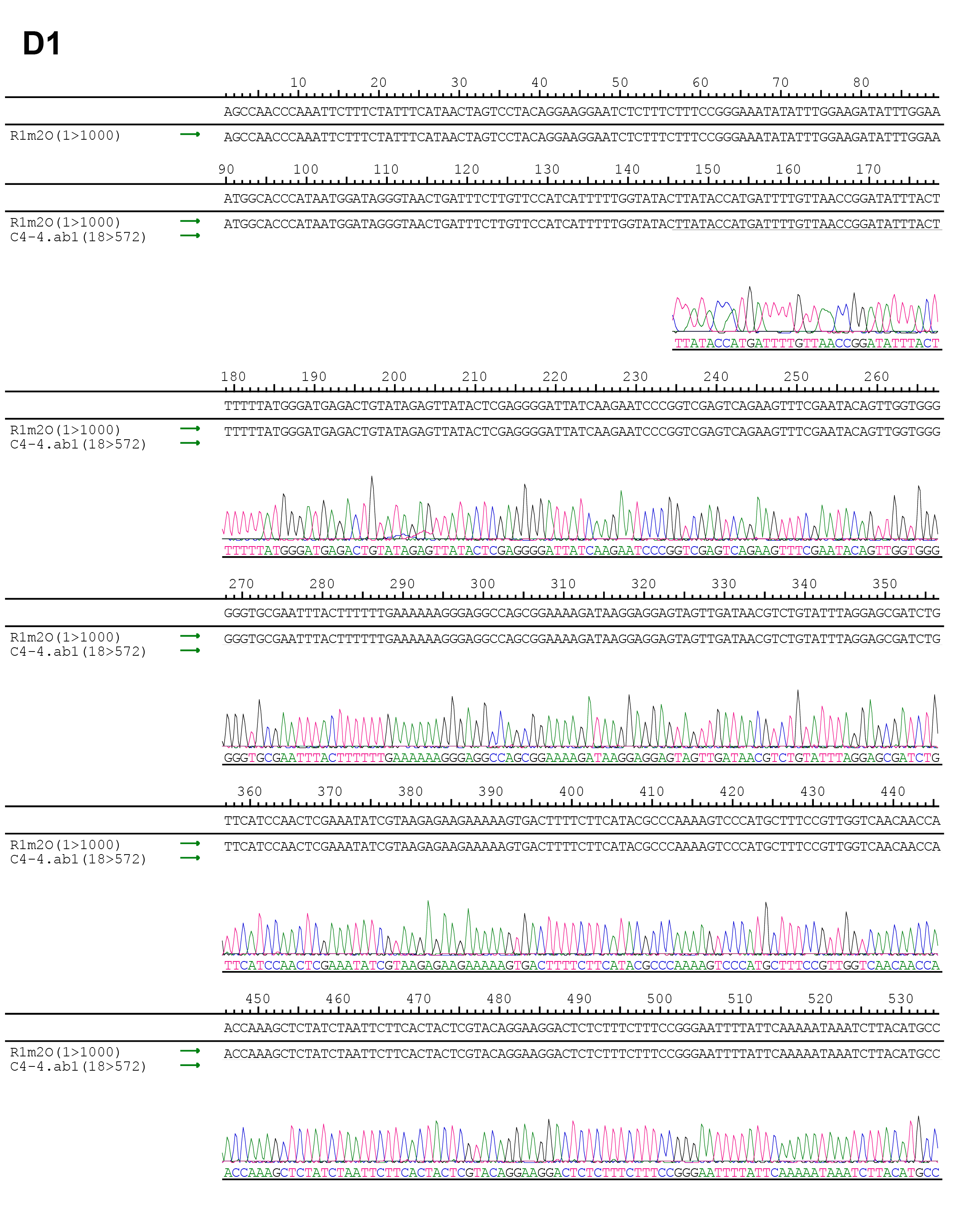


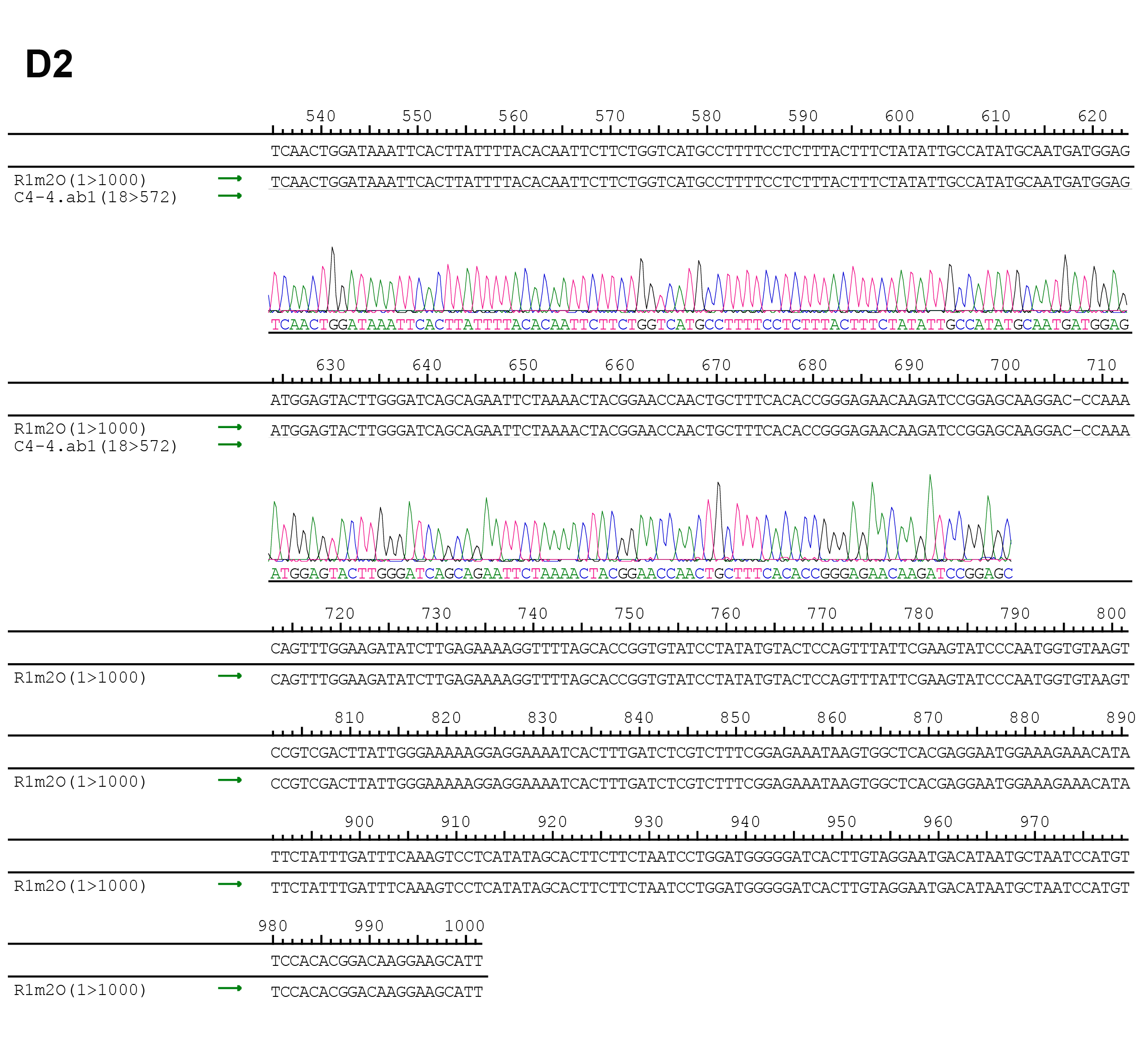


**Figure S3**. Boundary verification of repeat-mediated recombination (D: C4-R2)


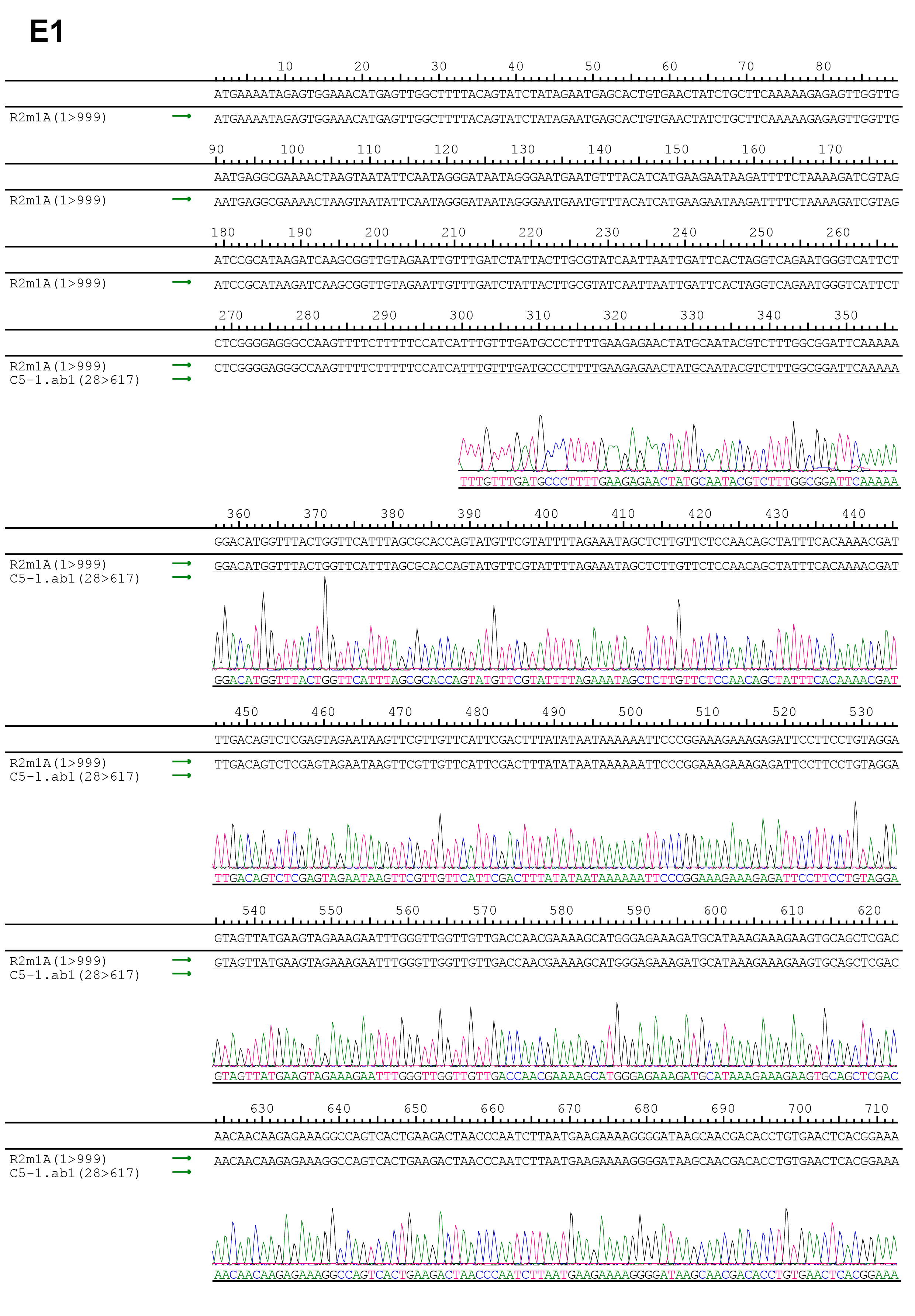


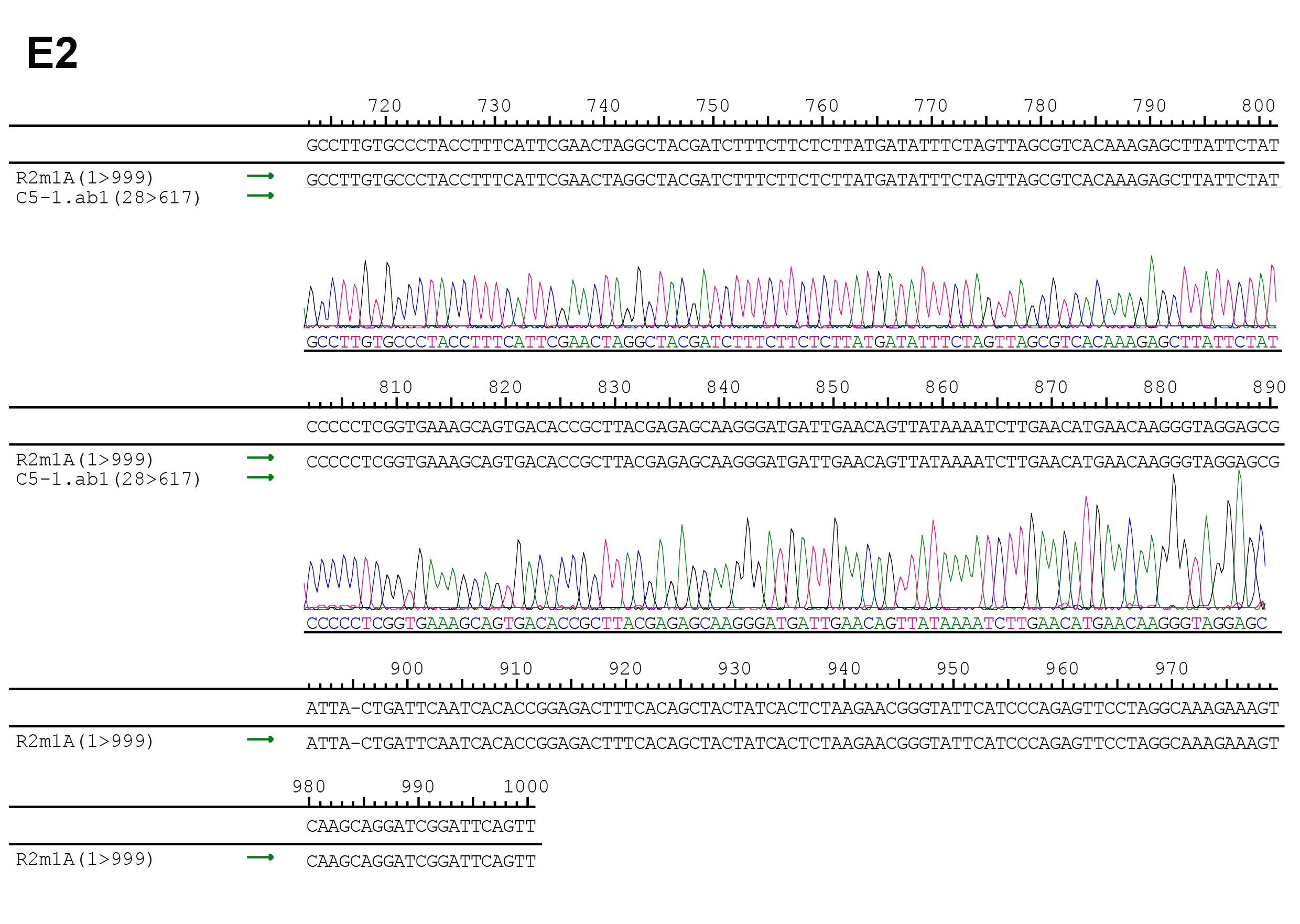


**Figure S3**. Boundary verification of repeat-mediated recombination (E: C5-F1)

**
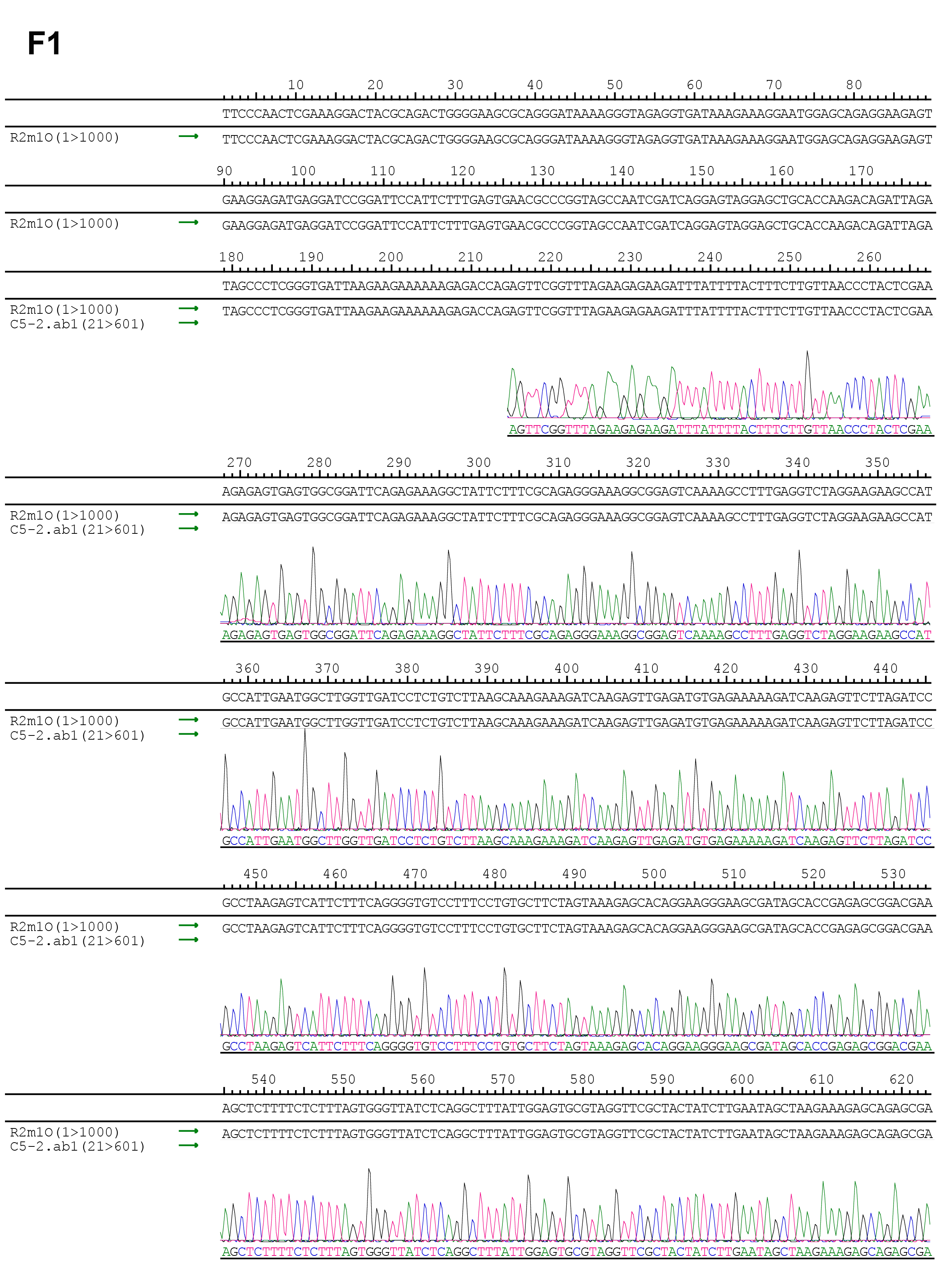
**

**
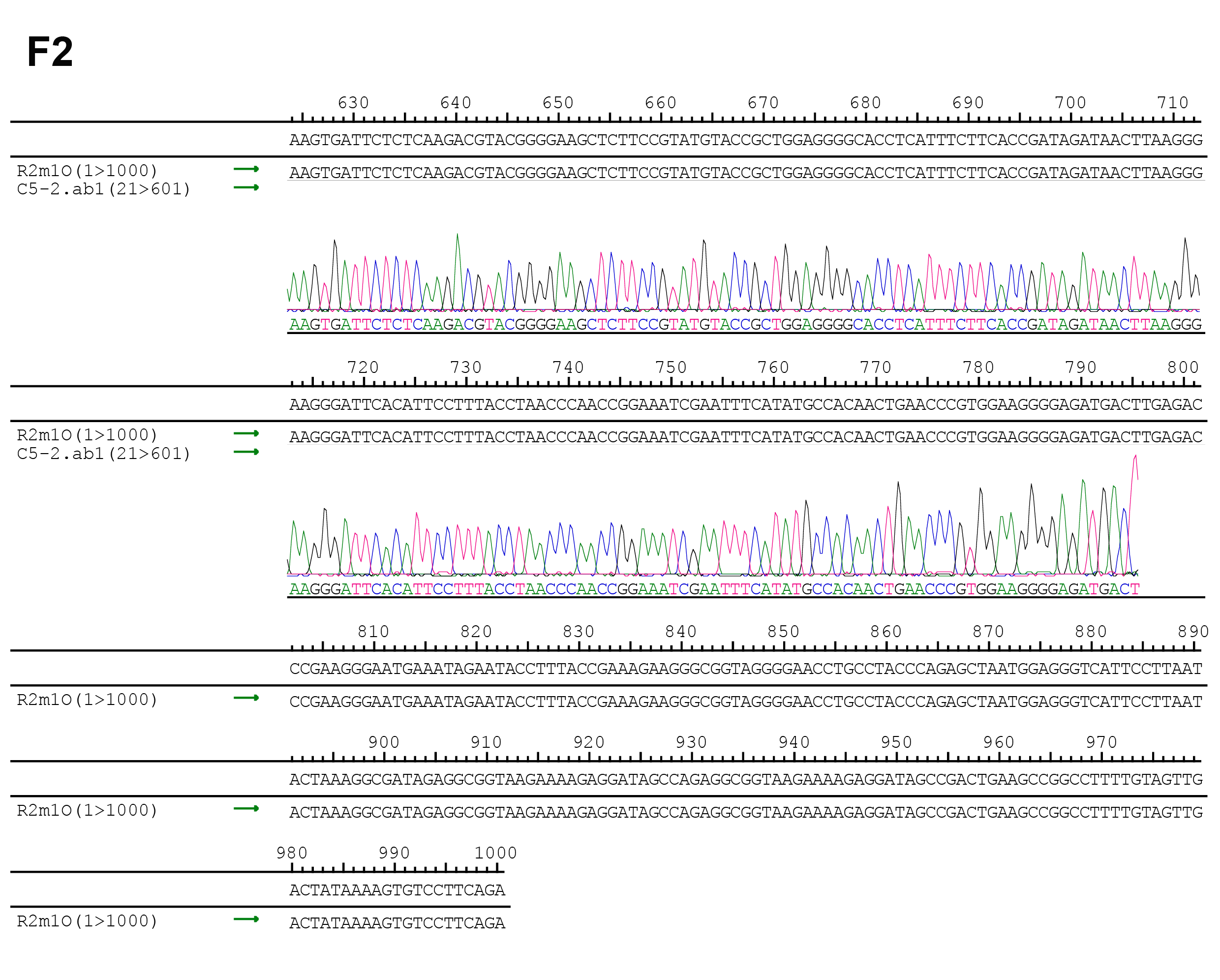
**

**Figure S3**. Boundary verification of repeat-mediated recombination (F: C5-R1)


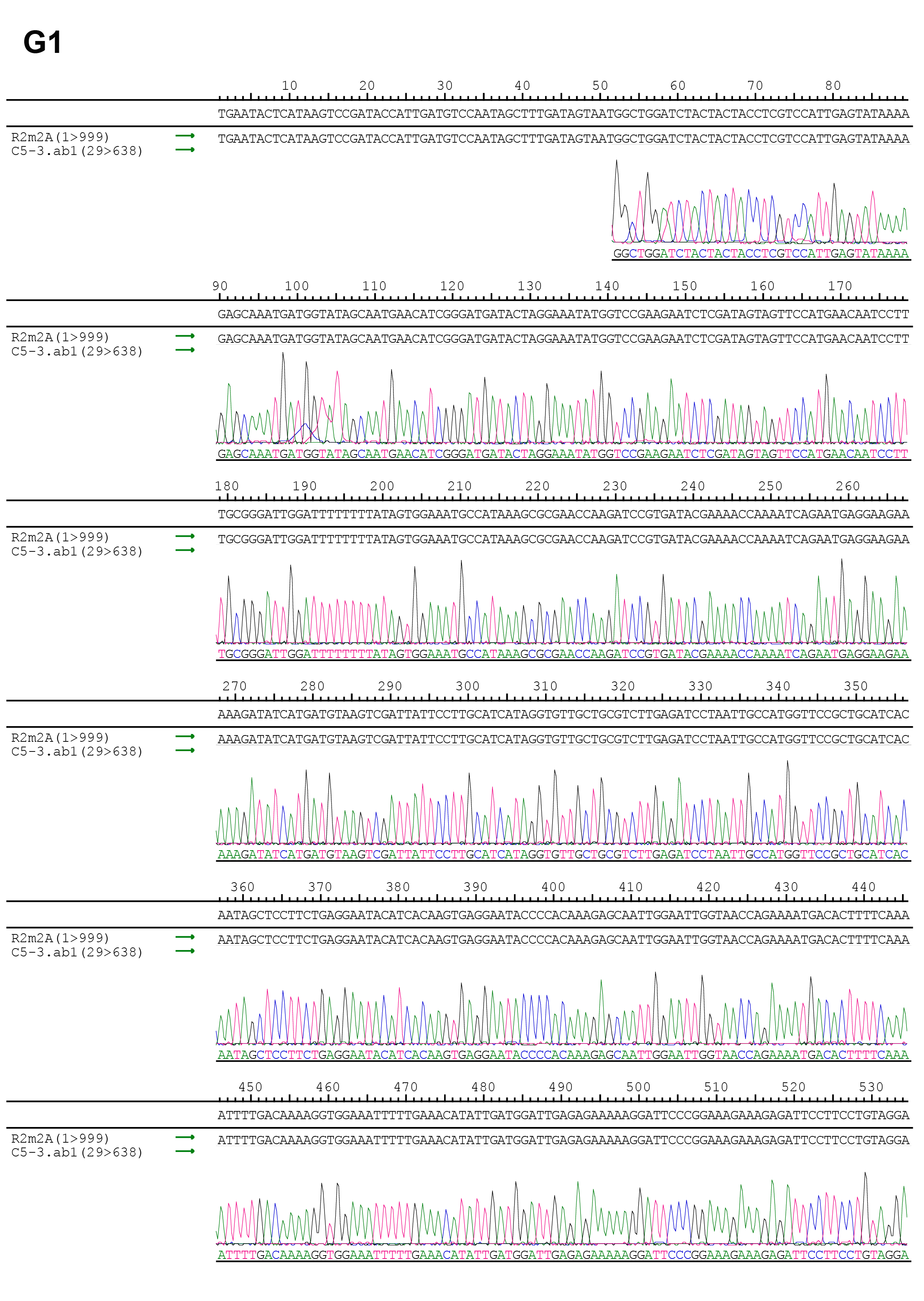


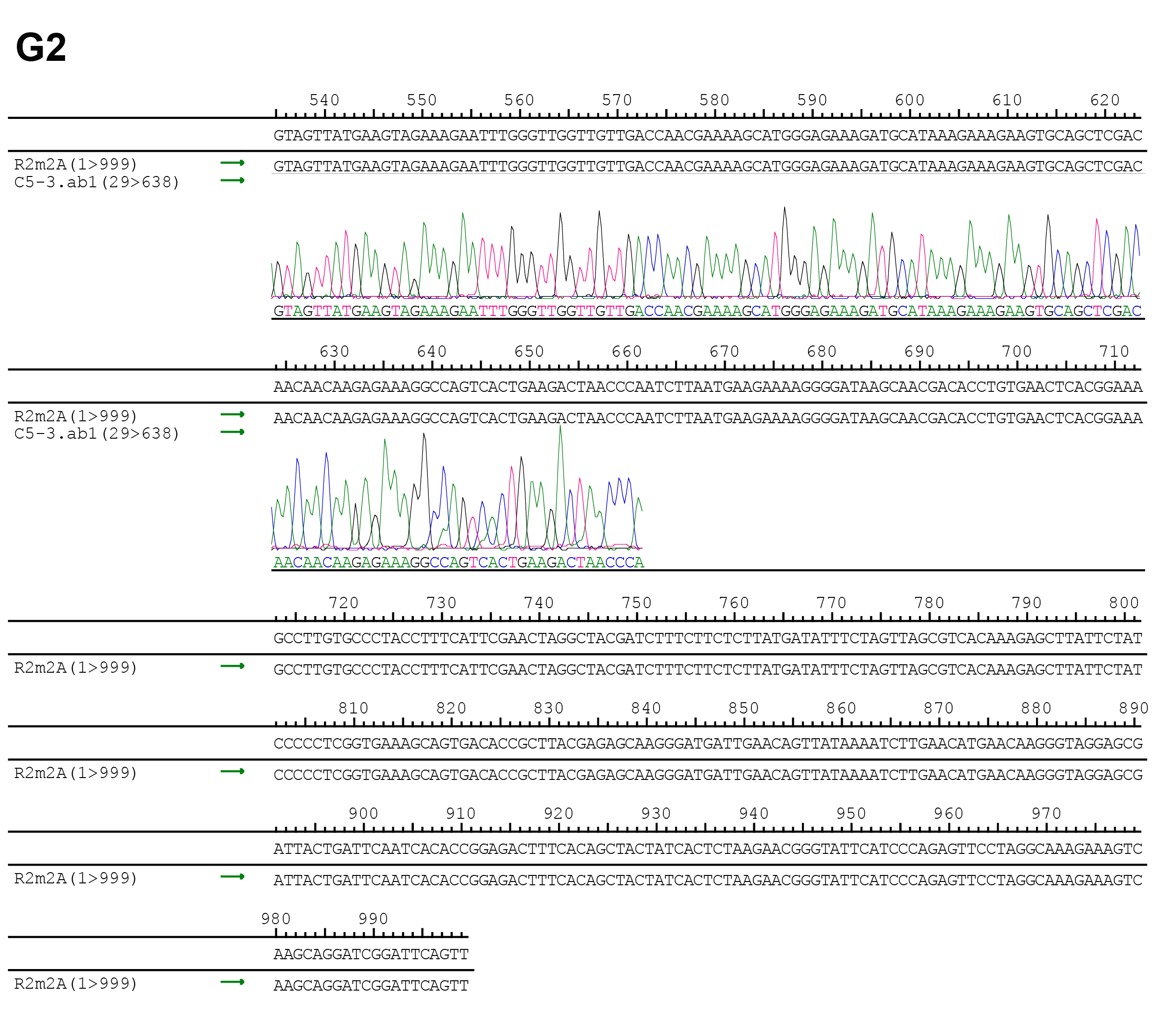


**Figure S3**. Boundary verification of repeat-mediated recombination (G: C5-F2)


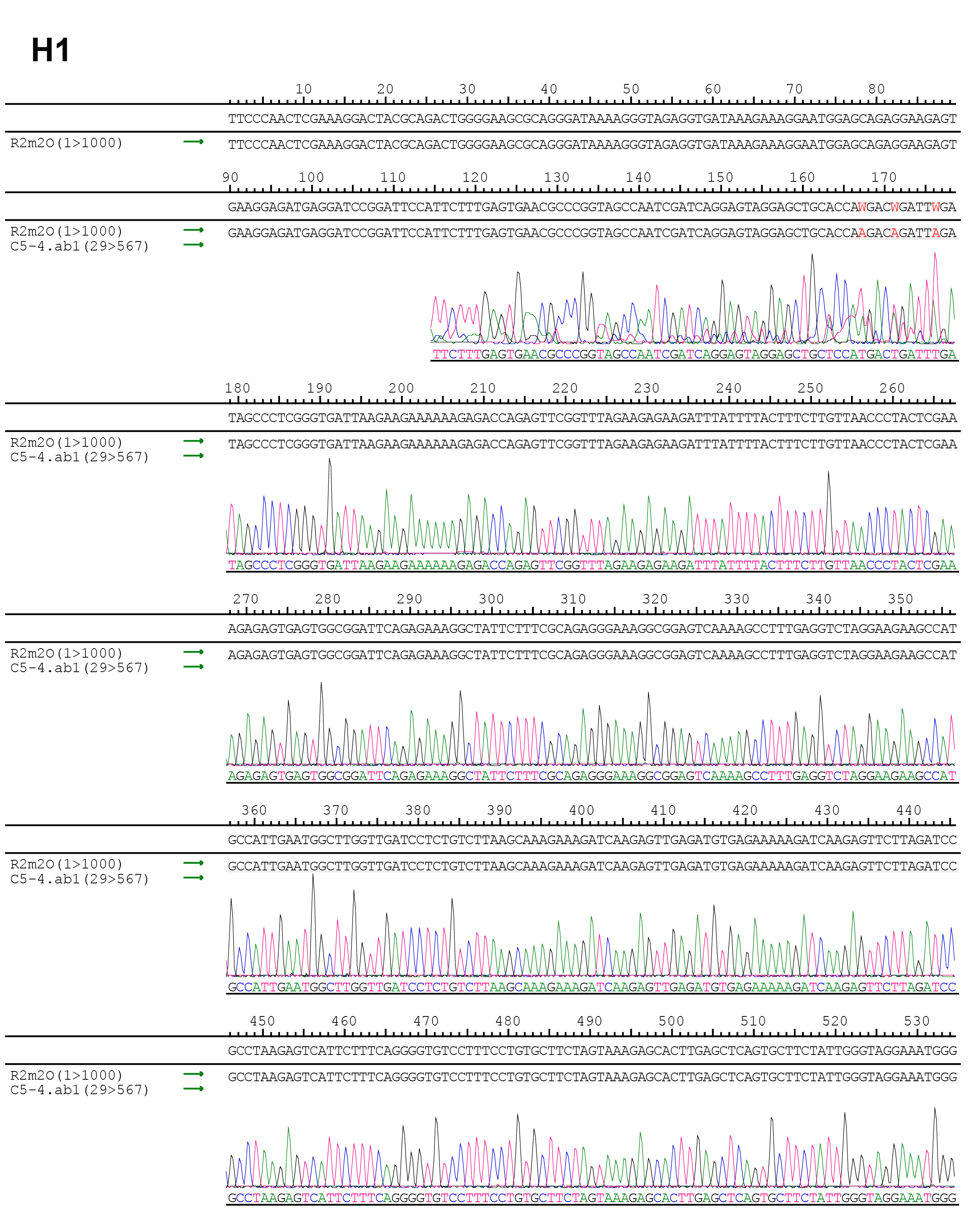


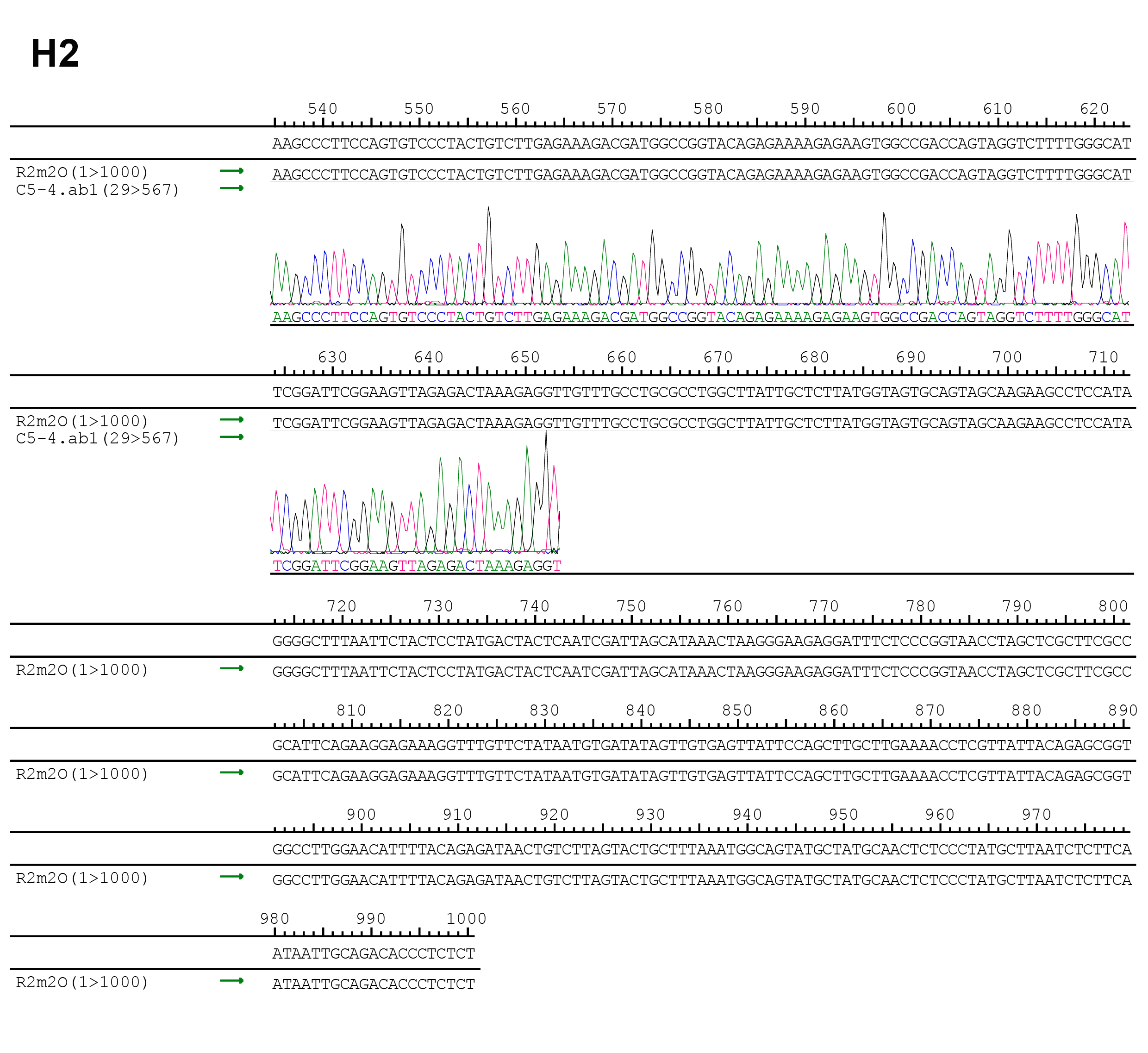


**Figure S3**. Boundary verification of repeat-mediated recombination (H: C5-R2)
