# Supplementary figures and images for: Complete mitochondrial genome of Syzygium samarangense reveals genomic recombination, gene transfer, and RNA editing events
Source: Front Plant Sci. 2024 Jan 9;14:1301164. doi: 10.3389/fpls.2023.1301164 (PMC10803518; doi:10.3389/fpls.2023.1301164)

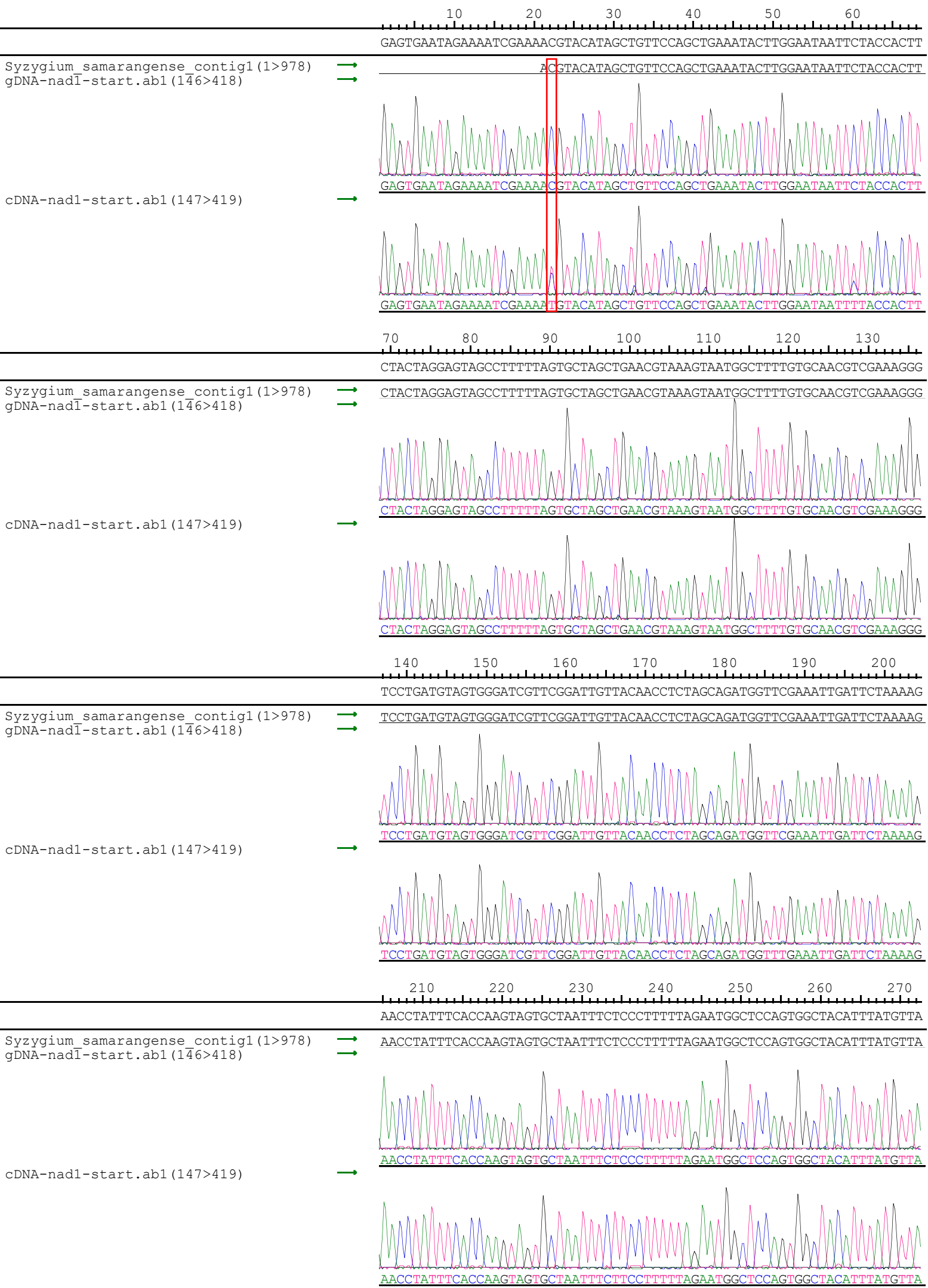

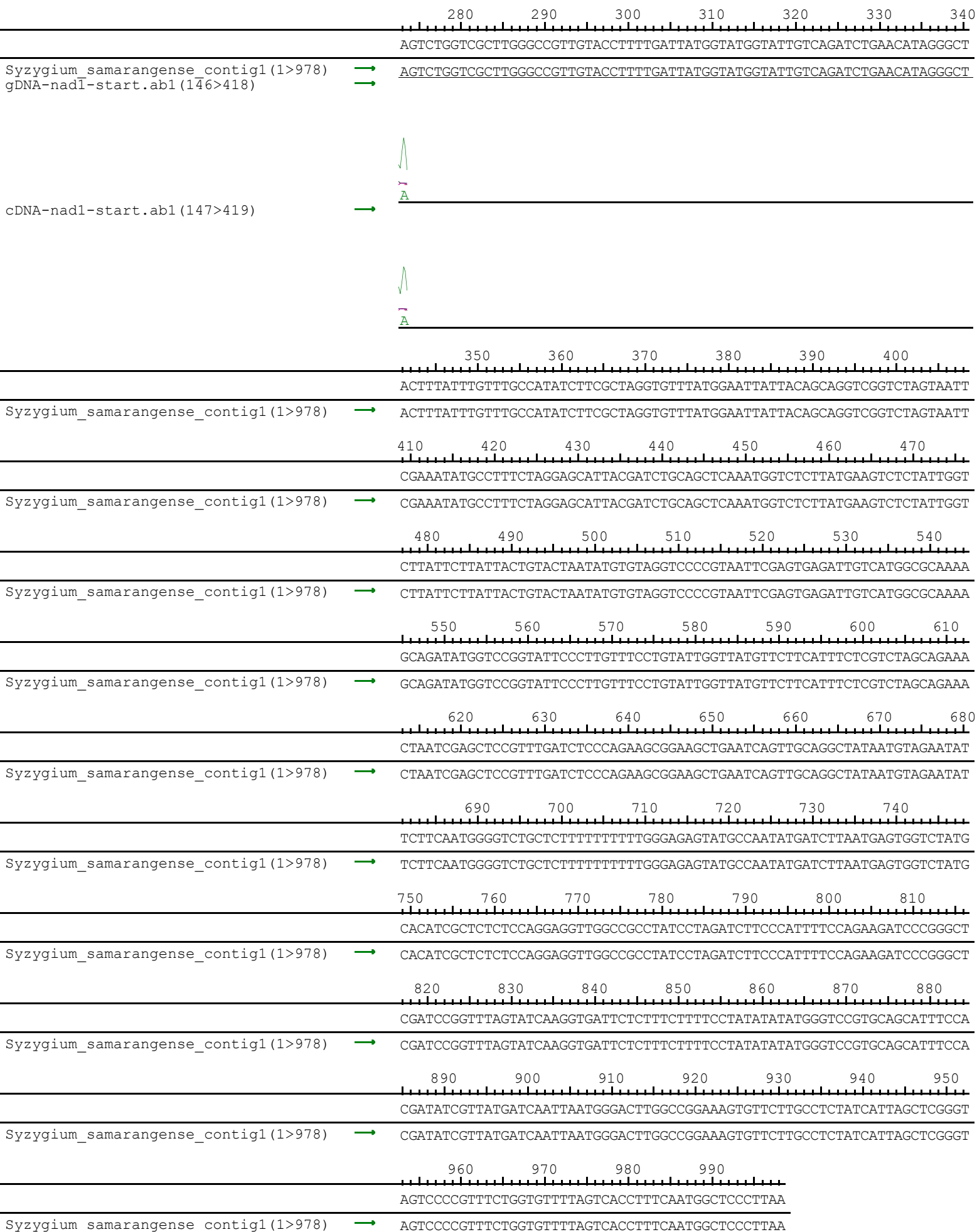

Supplement: Supplementary file 1 [file DataSheet_1.zip › Supplementary file 1/nad1-start.pdf]

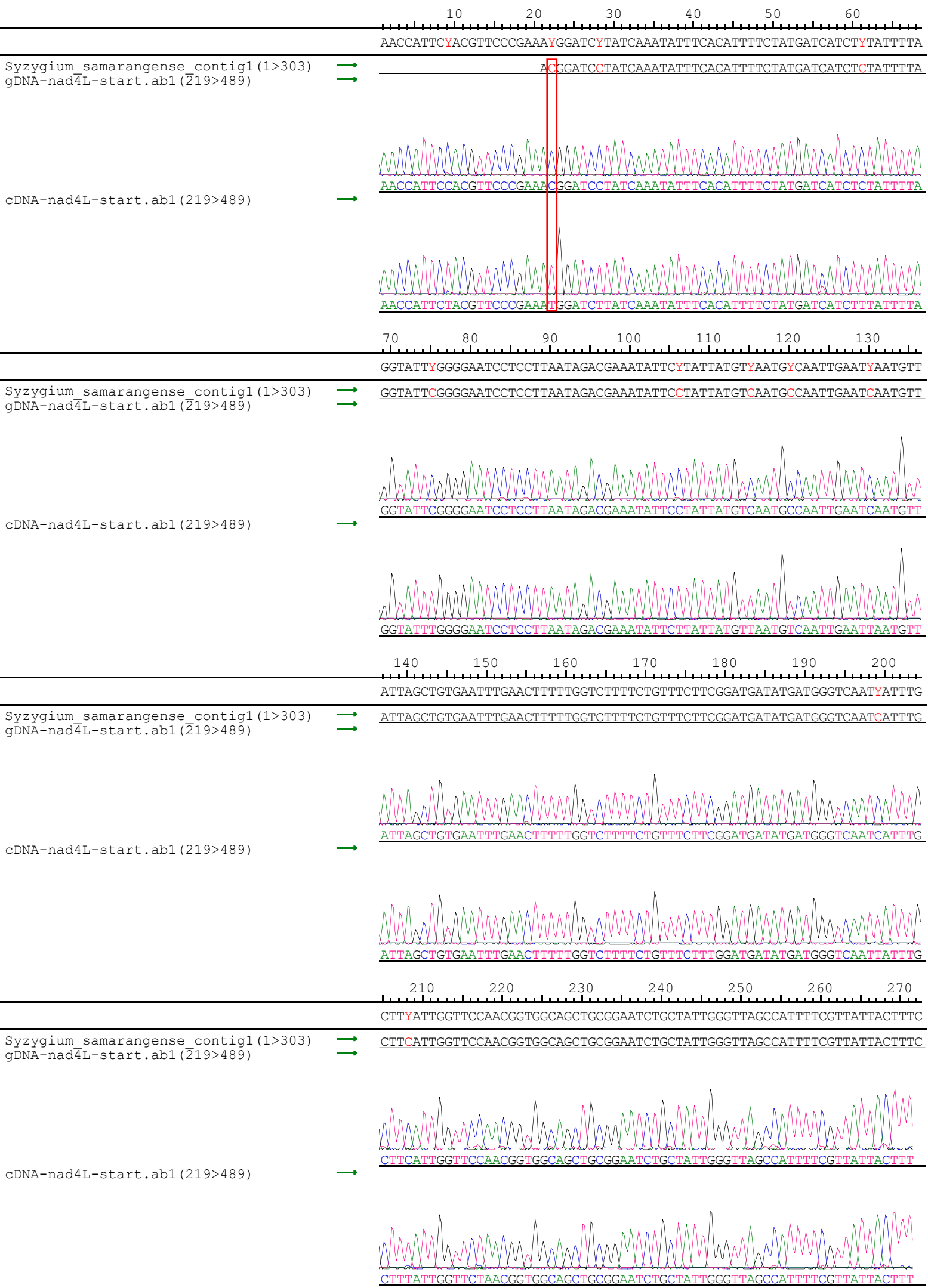

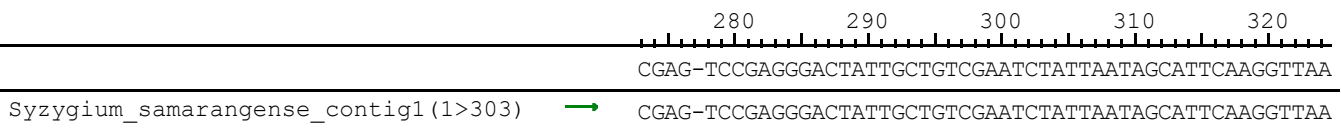

Supplement: Supplementary file 1 [file DataSheet_1.zip › Supplementary file 1/nad4L-start.pdf]

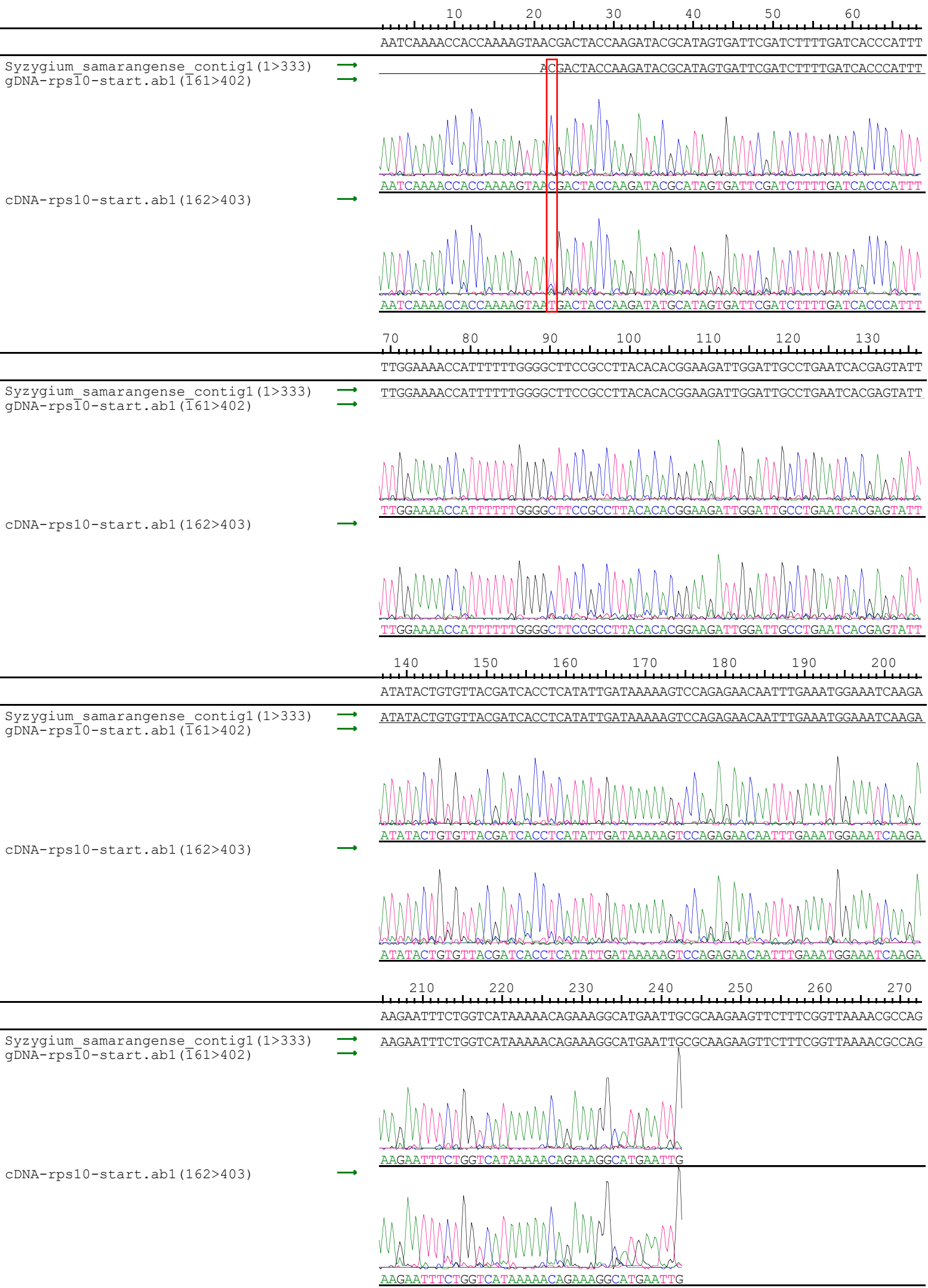

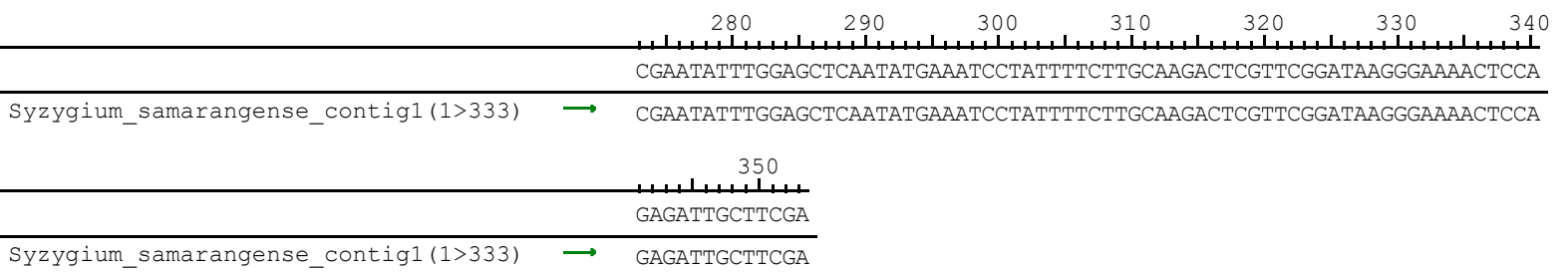

Supplement: Supplementary file 1 [file DataSheet_1.zip › Supplementary file 1/rps10-start.pdf]

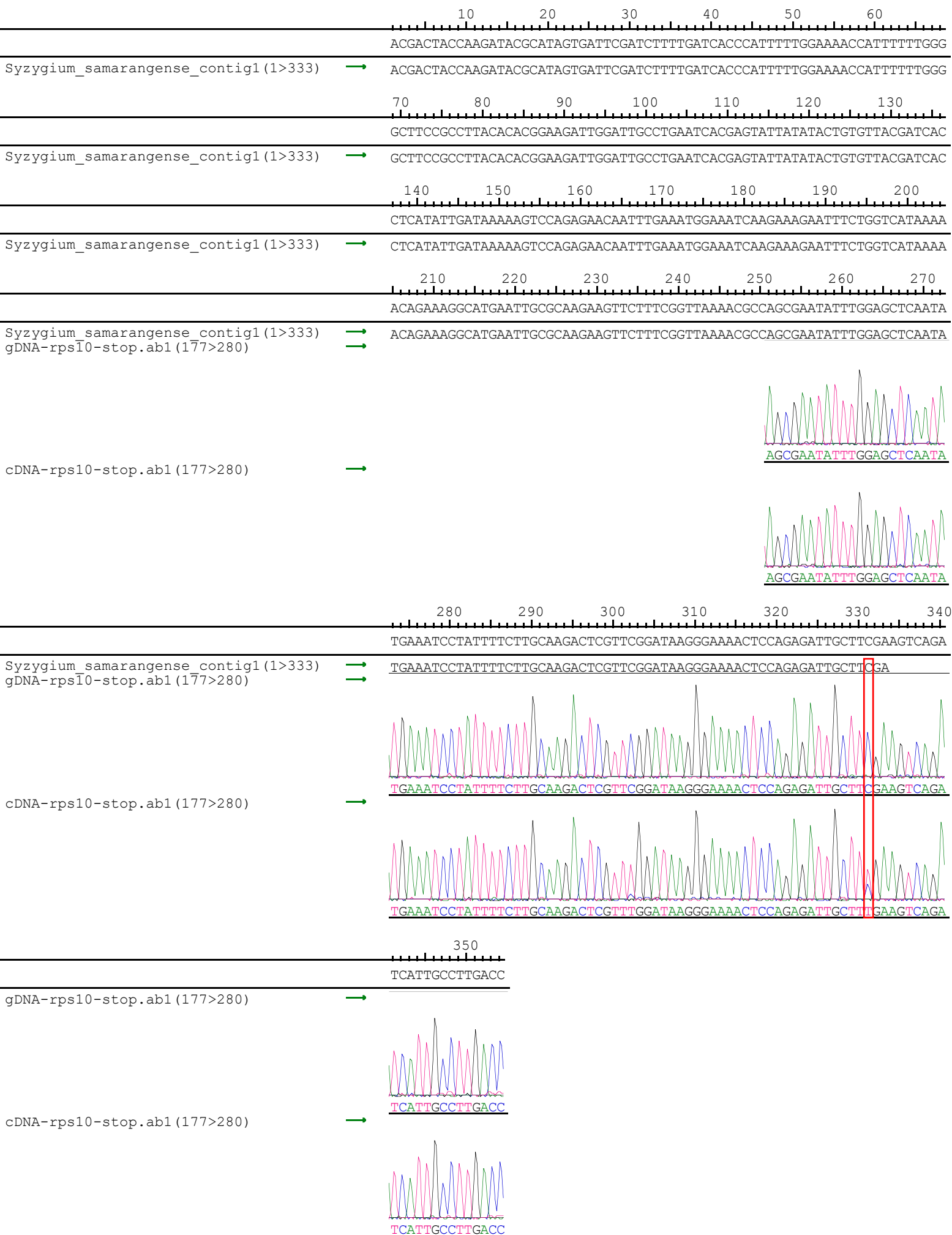

Supplement: Supplementary file 1 [file DataSheet_1.zip › Supplementary file 1/rps10-stop.pdf]
